# Safety and effectiveness of a novel neuroprotectant, KUS121, in patients with non-arteritic central retinal artery occlusion: An open-label, non-randomized, first-in-humans, phase 1/2 trial

**DOI:** 10.1371/journal.pone.0229068

**Published:** 2020-02-13

**Authors:** Hanako Ohashi Ikeda, Yuki Muraoka, Masayuki Hata, Eriko Sumi, Takafumi Ikeda, Takayuki Nakagawa, Hiroyasu Abe, Harue Tada, Satoshi Morita, Akira Kakizuka, Nagahisa Yoshimura, Akitaka Tsujikawa

**Affiliations:** 1 Department of Ophthalmology and Visual Sciences, Kyoto University Graduate School of Medicine, Kyoto, Japan; 2 Institute for Advancement of Clinical and Translational Science, Kyoto University Hospital, Kyoto, Japan; 3 Laboratory of Functional Biology, Kyoto University Graduate School of Biostudies & Solution Oriented Research for Science and Technology, Kyoto, Japan; Universita degli Studi di Firenze, ITALY

## Abstract

Kyoto University Substance (KUS) 121, an ATPase inhibitor of valosin-containing protein, is a novel neuroprotectant. We tested the safety and effectiveness of KUS121 in patients with acute central retinal artery occlusion (CRAO). We conducted an investigator-initiated, first-in-humans, phase 1/2 clinical trial. Nine patients with non-arteritic CRAO symptoms lasting for 4–48 h were enrolled. These patients received daily intravitreal injections of KUS121 for 3 days: 25 μg (low-dose) in the first three patients and 50 μg (high-dose) in the next six patients. The primary endpoint was the safety of the drug. As a secondary endpoint, pharmacokinetics was evaluated. Other key secondary endpoints were changes in best-corrected visual acuity (BCVA), measured using the Early Treatment Diabetic Retinopathy Study chart, visual field scores, and retinal sensitivities between baseline and week 12; and decimal BCVA at week 12. Administration of KUS121 did not result in serious adverse events. All nine patients (100%) showed significant improvement of BCVA. Average readable letter counts, visual field scores, and retinal sensitivities also improved. Decimal BCVA at week 12 was better than 0.1 in four patients (44%) and equal to or better than 0.05 in seven patients (78%). This first-in-humans clinical trial provides support for the safety and efficacy of intravitreal KUS121 injection. To substantiate the safety and effectiveness for patients with acute CRAO, further larger scale clinical studies will be needed.

## Introduction

The central retinal artery (CRA) perfuses the entire inner human retina; CRA occlusion (CRAO) leads to sudden profound loss of vision because the neuronal cells (e.g., ganglion cells) in the inner retina cannot process or transmit signals from the primary neurons, i.e., the photoreceptors. The annual incidence of this condition is reported to be 0.7–1.8 per 100,000 people [1–3]. The disease is classified into arteritic and non-arteritic types. In arteritic CRAO, the CRA is occluded due to inflammation-induced narrowing and requires prompt treatment with systemic steroids. In non-arteritic CRAO, the CRA is occluded by thrombosis or embolisms without arteritis. The degree of visual loss depends on the duration or severity of retinal ischemia and the presence of the cilioretinal artery, derived from choroidal flow, which perfuses the macula.

Studies using rhesus monkeys have reported that retinal ischemia lasting more than 4 h results in irreversible morphological and functional retinal damage [4]. Clinical trials of surgical removal of the embolus [5], local intra-arterial fibrinolysis [6, 7], and use of intravenous tissue-type plasminogen activator [8, 9] have been reported. Additionally, a meta-analysis investigating visual prognosis of CRAO patients has suggested that early systemic fibrinolytic therapy might result in a better visual prognosis [10]. Despite this, no effective treatment for improving visual outcomes in CRAO patients has been established to date [10].

It has been reported that neurons in the inner retina (e.g., retinal ganglion cells) of rhesus monkeys begin to degenerate 3–5 h after the start of retinal ischemia, and most of the neurons degenerate 16 h after ischemia commences [11]. In porcine experiments, degenerative changes in the retinal ganglion cells became prominent 5 h after retinal ischemia, and the die-off of retinal ganglion cells had ceased by 7 days after the bout of ischemia [12]. Another report, using rats, indicated that loss of the retinal ganglion cells mostly occurs between 6 and 24 h after retinal ischemia/reperfusion injury, and rarely occurs after 5 days [13]. The results of these reports suggest that death of the inner retinal neurons of patients with CRAO occurs mainly between 6 and 24 h after the onset and does not continue beyond 3–5 days after the onset. Once retinal ganglion cells are exposed to retinal ischemia, most of the retinal ganglion cells die, mainly due to endoplasmic reticulum (ER) stress even after reperfusion of the inner retina [14].

We had developed small molecules termed Kyoto University Substances (KUSs) [15] as novel agents against neuronal damage, as observed in neurodegenerative diseases. KUSs specifically inhibit the ATPase activity of valosin-containing protein (VCP) [16], the most abundant, ubiquitously expressed, soluble ATPase, without inhibiting other cellular VCP functions [15]. We therefore term KUSs as VCP modulators. KUSs have been shown to maintain cellular ATP levels, function as intracellular ATP regulators, and consequently suppress ER stress and cell death under various cell death-inducing insults, both in vitro and in several pathological conditions in vivo [15, 17–20]. We have shown that KUSs can mitigate retinal pathologies in animal models of retinitis pigmentosa [15, 17], glaucoma [18], age-related macular degeneration [20], and retinal ischemia [19]. Furthermore, KUS121 has been shown to be one of the most potent neuroprotective KUSs and has not shown any obvious safety issues in these animal experiments. Additionally, in rats with ischemic retinal injuries, either systemic or intravitreal administration of KUS121 suppressed death of the retinal ganglion cells, as well as subsequent deterioration of visual functions [19].

Based on findings from the aforementioned animal experiments, patients with acute-phase CRAO are expected to exhibit sudden loss of visual acuity and fields due to loss of function of the neurons in the ischemic inner retina. Within 1 week, the neurons exposed to ischemia in the inner retina are expected to degenerate, which may cause profound and permanent visual detriment observed in these patients. If KUS121 is supplied to the inner retina before the neurons die and suppresses their death, the surviving neurons should recover their function following reperfusion of the CRA or diffusion of the choroidal blood flow. Subsequently, the patients should recover their vision. Therefore, we decided to examine whether KUS121 could act as a novel neuroprotectant for patients with acute CRAO.

Intravitreal injection is an established drug delivery route in ocular diseases [21]. Intravitreal injection enables immediate and adequate delivery of agents with fewer systemic influences. Furthermore, this route of delivery is expected to remain unaffected by the loss of blood flow. Therefore, we selected intravitreal injection as the administration route of KUS121 for patients with CRAO. Here, we report the results from the first-in-humans clinical trial (phase 1/2 study) conducted to assess the safety, tolerability, pharmacokinetics, and effectiveness of daily intravitreal injections of KUS121 for 3 days in patients with non-arteritic acute CRAO.

## Methods

### Study design and participants

This study was an investigator-initiated, phase 1/2, dose-escalation trial conducted at Kyoto University Hospital. The aim of this study was to test the safety, tolerability, pharmacokinetics, and efficacy of daily intravitreal injections of KUS121, for 3 days, in patients with non-arteritic central retinal artery occlusion. It adhered to the tenets of the Declaration of Helsinki and the Ministerial Ordinance on Good Clinical Practice for Drugs and was approved by an ethics committee (C1201, Kyoto University Graduate School and Faculty of Medicine, Ethics Committee), an Institutional Review Board (K028, Kyoto University Hospital Institutional Review Board) in Kyoto University Hospital, and the Pharmaceuticals and Medical Devices Agency of Japan. This trial is registered in the University Hospital Medical Information Network clinical trial registry (registration identification number is UMIN000023979, date of first registration: 8/9/2016).

We recruited consecutive nine patients who visited Kyoto University Hospital in order with non-arteritic CRAO, aged 20 years or more, exhibiting symptoms lasting for 3–48 h after onset, to exclude patients with transient CRAO, after obtaining written informed consent. We diagnosed CRAO based on a cherry-red spot with retinal cloudiness due to swelling; narrowing, or disruption of retinal arteries; and blocking or retardation of blood flow in retinal arteries, as examined using fluorescent angiography. Sample size was determined from the viewpoint of feasibility, based on the annual incidence of the disease and the intention to conduct a 1-year study. The Kyoto district has a population of 2.61 million and the incidence of CRAO is reported to be 0.7–1.8 per 100,000 people per year [1–3], meaning that about 20 patients are expected to develop CRAO annually. Decimal best-corrected visual acuity (BCVA) was required to be equal to or lower than 0.1 and be better than hand motion (HM). Male patients were required to agree to use effective contraception from the first injection until 4 weeks after the last injection. Female patients were required to be either post-menopausal or surgically infertile, because a reproduction toxicity test had not been performed. Inclusion criteria for systemic conditions were: hemoglobin, 10 g/dL or higher; white blood cell count, 2,000/μL or greater; platelet count, 100,000/μL or greater, aspartate aminotransferase levels, 50 IU/L or lower; alanine aminotransferase levels, 50 IU/L or lower; total bilirubin levels, 1.5 mg/dL or lower; and creatinine levels, 1.5 mg/dL or lower. Exclusion criteria for ocular conditions were as follows: eyes with uncontrolled intraocular pressure, despite medication; retinal vein occlusion; abnormality in the macula, except those consistent with CRAO; or opacity in the lens or the vitreous, such that the fundus was not visible. Patients with a fellow-eye BCVA of 0.1 or lower, or loss of half or more of the visual field in isopter I4e in Goldmann perimetry, with bilateral onset of CRAO, or with systemic use of immunosuppressant or steroid medications, were excluded.

### Procedures

The patients received intravitreal KUS121 injections (mainly by Y.M. and M.H.) at a Kyoto University Hospital ophthalmology ward. The dose of each intravitreal KUS121 injection was 25 μg/100 μL in the low-dose group (first three patients) and 50 μg/100 μL in the high-dose group (the next six patients). The doses were determined based on data of time-dependent intravitreal drug concentration obtained using macaques (unpublished data) and the 90% ATPase inhibitory concentration (964 nM) and half-maximal inhibitory concentration (109 nM) of KUS121 (ref. [15] and unpublished data). The optimal dose for clinical use was speculated to be 50 μg/eye/injection. An unequal distribution (3 patients for low dose and 6 patients for high dose) was set, as we believed it was most important to improve the statistical power for the detection of significant effects at the highest dose. Patients received injections on the day of enrolment (defined as day 1), day 2 (24 h after the first injection), and day 3 (24 h after the second injection). If the intraocular pressure immediately after the injection was ≥ 5 mmHg higher than before the injection, paracentesis was performed to lower the intraocular pressure to pre-treatment levels. During the study, patients did not receive other possible treatment options for acute CRAO (e.g., ocular massage, sublingual isosorbide dinitrate, systemic pentoxifylline, inhalation of 10% carbon dioxide, and hyperbaric oxygen), although we did not exclude patients who had received one or more of these treatments prior to inclusion in the study. Ancillary care for CRAO was not performed in this study, except for treatments started before inclusion in the study (e.g., anti-coagulant medications, anti-hypertensive agents, etc.).

### Assessment of safety and effectiveness

BCVA was tested using both a decimal visual acuity chart (Randolt rings) and the Early Treatment Diabetic Retinopathy Study (ETDRS) charts (alphabet letters) at baseline and in weeks 2, 4, 8, and 12 after treatment. When no letter could be read on the decimal visual acuity chart or ETDRS chart, the BCVA was classified into counting fingers, HM, light perception (LP), or no light perception (NLP). Logarithm of the minimum angle of resolution (logMAR) for counting fingers, HM, LP, or NLP were assigned as 2.6, 2.9, 3.1, or 3.4, respectively, according to a previous report [22]. Goldmann perimetry tests were performed at baseline and in weeks 2, 4, and 12. Retinal sensitivity was measured using Micro Perimeter-3 (MP-3; Nidek, Aichi, Japan) at baseline and in weeks 2, 4, and 12. Optical coherence tomography (OCT) was performed using a Spectralis HRA+OCT (Heidelberg Engineering, Heidelberg, Germany) at baseline, on days 2, 3, and 4; and in weeks 2, 4, 8, and 12 after treatment. Fluorescein angiography was performed at baseline and on day 6 after treatment.

Plasma KUS121 concentration was measured at Shin Nippon Biomedical Laboratories (Wakayama, Japan) using high-performance liquid chromatography/tandem mass spectrometry at 2 and 4 h after the first injection (day 1); immediately before, and at 2 and 4 h after the second injection (day 2); and immediately before, and at 2, 4, and 24 h after the third injection (day 3). Concentrations below 0.05 ng/mL, the limit of determination, were considered zero.

The primary endpoint of this study was to assess the safety of KUS121 intravitreal injections, including adverse events (all unfavorable events, even if they are known to occur in the disease course) and side effects (all adverse events for which the possibility of being drug-related could not be excluded) during the 12 weeks of observation. We defined a serious adverse events according to the Ministerial Ordinance on Good Clinical Practice guidelines: (1) Results in death, (2) Is life threatening, (3) Requires inpatient hospitalization or prolongation of existing hospitalization for the treatment, (4) Results in persistent or significant disability/incapacity, (5) Leads to a congenital anomaly/birth defect, and (6) Is an important medical condition not specified above. The key secondary endpoints included assessment of the pharmacokinetics of KUS121, visual acuity, visual field, and retinal sensitivity, to evaluate drug effectiveness. The BCVA obtained using the ETDRS chart at each examination was presented in logMAR values and readable-letter counts. For comparison with the reported natural course of the disease [23], the proportions of patients with decimal BCVA of 0.02 or better, 0.05 (the borderline value of blindness defined by World Health Organization [24]) or better, and 0.1 or better at week 12, were also evaluated. The visual field area at week 12 was evaluated with V4e, I4e, and I2e isopters of Goldmann perimetry using Photoshop CC software (Adobe Inc., San Jose, CA, USA). Visual field [25] and Esterman disability [26] scores were also assessed in a blinded manner (by AH). Retinal sensitivity at week 12 was evaluated as the average of eight central points (at 5° from the fovea), eight outer points (at 10° from the fovea), and eight outermost points (at 15° from the fovea), using MP-3 microperimetry. Retinal sensitivity data in one patient (#6) could not be acquired due to poor fixation, stemming from poor visual acuity and a narrowed visual field at baseline. Changes in each value between baseline and week 12 were also evaluated. All the instruments were regularly maintained and validated.

### Descriptive analysis

All the data were collected at the data center at the Kyoto University hospital. Analyses of safety and effectiveness were based on a June 2018 database-lock. The safety analyses included all nine patients who were enrolled in this study and received trial treatment, and the incidence of adverse events in the patients in each group was evaluated from baseline to week 12. The rate of incidence and the 95% confidence intervals were calculated. Plasma KUS121 concentrations at each time point were reported descriptively (mean and standard deviation for each group), and maximum drug concentration (C_max_), time of occurrence of C_max_ (T_max_), and areas under the concentration–time curve from time zero to 24 h, 24 to 48 h, 48 to 72 h, and 0 to 72 h were calculated by a non-compartmental method (Phoenix WinNonlin, version 6.3, Princeton, NJ, USA.) The effectiveness analyses included all nine patients (who received all scheduled trial treatments). Descriptive statistic values (mean, standard deviation, and minimum and maximum values for each group) and 95% confidence interval were calculated for each group. For continuous variables, the averages were used to calculate 95% confidence intervals based on the t-statistic, while for binary variables, proportions were used to calculate Clopper-Pearson 95% confidence intervals. All analyses, except for pharmacokinetics, were performed using the SAS statistical software, version 9.4 (SAS Institute, Cary, NC, USA).

## Results

**Fig 1:**
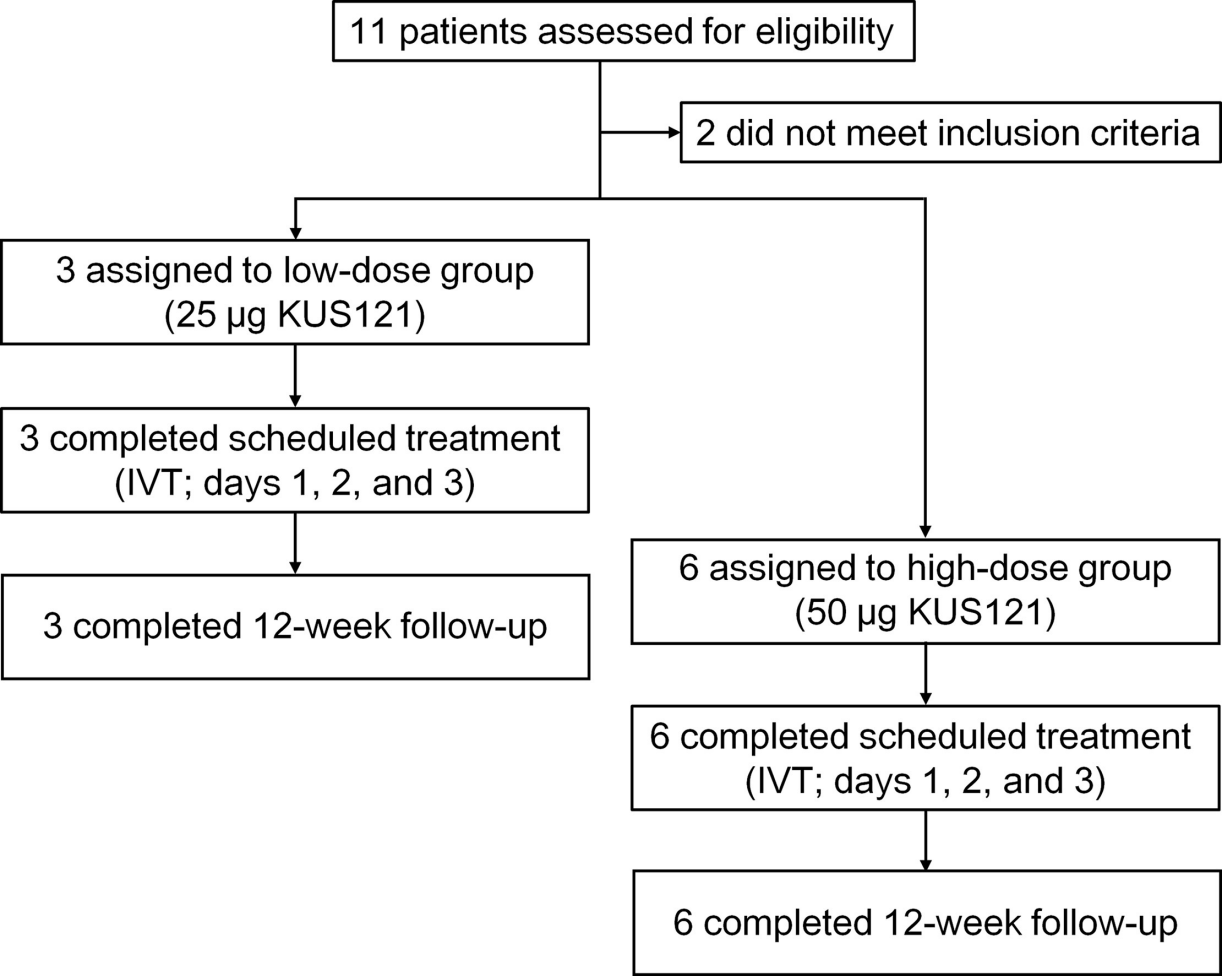
Trial profile. IVT: intravitreal injection of KUS121, KUS: Kyoto University Substance.

Between November 22, 2016, and December 5, 2017, 11 patients with non-arteritic acute CRAO were assessed for eligibility, and nine patients were recruited for the study (Fig 1 and Table 1). Of the 11 patients, one was excluded because of voluntary withdrawal of consent, and the other was excluded due to a high total bilirubin level. The first three patients were assigned to the low-dose group, and the subsequent six patients were assigned to the high-dose group. All nine enrolled patients completed the follow-up, and their data were included in the analysis. There were no major protocol deviations from the study plan. The mean age (average ± standard deviation) was 60.0 ± 22.3 years for the low-dose group, and 73.7 ± 18.4 years for the high-dose group.

**Table 1:** Baseline characteristics of systemic condition of patients.

Patient No.	Age (years)	Sex	BMI	Systemic complications/history					Smok-ing	BI	Pre-treatment before initial visit
				DM	HT	HL	HD	Others			
**Low-dose group**											
1	78	M	25.0	+		+	+				
2	67	M	26.1	+	+		+	Hyperuricemia	+	1200	Topical latanoprost
3	35	M	23.2		+			Renal dysfunction	+	200	Ocular massage
**High-dose group**											
4	72	M	23.3	+	+	+					Ocular massage
5	88	F	23.2		+		+	Aortic aneurysm			Topical brinzolamide
6	82	F	25.8		+		+				
7	77	M	24.7		+	+		Hyperuricemia	+	800	Paracentesis, ocular massage, mannitol, alprostadil
8	38	M	22.9					The right (affected eye) OA originates from the MMA	+	240	Ocular massage
9	85	M	18.1			+	+	Carotid artery stent	+	175	

M: male, F: female, BMI: body mass index, DM: diabetes mellitus, HT: hypertension, HL: hyperlipidemia, HD: heart diseases, OA: ocular artery, MMA: middle meningeal artery, BI: Brinkman Index = (number of cigarettes/day) × duration of smoking

Baseline characteristics of the ocular condition of the affected (objective) eye are summarized in Table 2. At baseline, the mean BCVA measured using ETDRS chart (logMAR and readable letter counts) was 2.46 ± 0.76 and 1.3 ± 2.3 letters for the low-dose group, and 1.97 ± 0.62 and 3.7 ± 3.5 letters for the high-dose group, respectively. The cilioretinal artery was present and its patency was judged based on fluorescein angiography in three patients (one from the low-dose group and two from the high-dose group). In six patients, pre-treatments, including paracentesis, ocular massage, and/or medications to lower intraocular pressure, had been performed. In all nine patients, three intravitreal injections of KUS121 (25 μg/100 μL in the low-dose group and 50 μg/100 μL in the high-dose group) were administered according to the study protocol. Time interval between the first intravitreal injection and (estimated) onset was 19.6 ± 8.0 h for the low-dose group and 27.6 ± 15.1 h for the high-dose group.

**Table 2:** Baseline characteristics of the objective eyes.

Patient No.	Affected eye	BCVA			IOP (mmHg)	Lens status	Intraretinal transit time (s)	Presence and patency of CRA	Time from onset to first IVT (h)
		Decimal notation	LogMAR (ETDRS)	ETDRS letter count (letters)					
**Low-dose group**									
1	R	0.06	1.58	4	16	IOL	90	-	26.0
2	R	HM	2.9	0	10	NS2	> 600	-	22.1
3	L	HM	2.9	0	7	Clear	183	+	10.7
**High-dose group**									
4	R	0.1	2.6	0	15	NS2	131	+	10.5
5	R	0.1	1.64	3	10	IOL	9	-	33.2
6	R	HM	2.9	0	10	NS2	225	-	38.6
7	L	0.1	1.58	6	6	NS2	> 600	-	29.8
8	R	0.09	1.46	9	12	NS1	> 600	+	8.3
9	L	0.02	1.62	4	8	NS3	44	-	45.4

BCVA: best-corrected visual acuity, HM: hand motion, logMAR: logarithm of the minimum angle of resolution, ETDRS: Early Treatment Diabetic Retinopathy Study, IOP: intraocular pressure, IOL: intraocular lens, NS: nuclear sclerosis of Emery-Little classification, Intraretinal transit time: the time between start of fluorescence staining in the retinal arteries and end of fluorescence filling in the retinal veins, CRA: cilioretinal artery, IVT: intravitreal injection. *Patients with 0.1 decimal BVCA had different logMAR values determined with ETDRS charts. This was probably because visual acuity tests are subjective and may fluctuate, especially in patients with a sudden decline in visual field.

There were no serious adverse events in either group. In all nine patients, intraocular pressure increased immediately after the intravitreal injection of 100 μL KUS121 solution, which was immediately decreased to the normal range by paracentesis (see Discussion). Corneal epithelial damage and conjunctival hemorrhage were observed as mild adverse events related to the procedure of intravitreal injections. As other ocular adverse events, macular edema with cystoid spaces and iris neovascularization were observed in the low-dose group. Foveal retinal detachment, macular edema with cystoid spaces, and recurrent retinal ischemia, because of re-occlusion of the CRA, were observed in the high-dose group. However, the patients gradually recovered from corneal epithelial damage, conjunctival hemorrhage, macular edema, foveal retinal detachment, and exacerbation of retinal ischemia without any further treatment. Iris neovascularization was eliminated after pan-retinal photocoagulation. Side effects that were undeniably associated with KUS121 are listed in Table 3.

**Table 3:** Side effects that were undeniably associated with KUS121.

	Low-dose group		High-dose group	
	N (%)	95% CI	N (%)	95% CI
Total	2 (66.7)	9.4, 99.2	1 (16.7)	0.4, 64.1
Iris neovascularization	1 (33.3)	0.8, 90.6	0 (0.0)	0.0, 45.9
Macular edema with cystoid spaces	1 (33.3)	0.8, 90.6	1 (16.7)	0.4, 64.1
Worsening of retinal ischemia	0 (0.0)	0.0, 70.8	1 (16.7)	0.4, 64.1

CI: confidence interval

Clopper-Pearson 95% confidence intervals were calculated for the rate of each side effect.

Plasma concentrations of KUS121 reached maximum levels 2 h after intravitreal injections, except on day 3 in the low-dose group. Maximal concentration of KUS121 (C_max_) on day 1 was 0.0721 ± 0.012 ng/mL in the low-dose group and 0.166 ± 0.106 ng/mL in the high-dose group (Tables 4 and 5). The concentrations decreased to sub-detection level 24 h after injection (immediately before the second injection). The plasma concentrations of KUS121 on days 2 and 3 were similar to that on day 1.

**Table 4:** Plasma concentrations of KUS121.

		Concentrations of KUS121 (ng/mL)								
		Day 1		Day 2			Day 3			
		2 h	4 h	Pre	2 h	4 h	Pre	2 h	4 h	24 h
Low-dose group	Average	0.0721	0.0342	0	0.0665	0.0371	0	0.0676	0.0690	0
	SD	0.0120	0.0296	0	0.0046	0.0321	0	0.0201	0.0258	0
High-dose group	Average	0.166	0.0635	0	0.221	0.124	0	0.147	0.107	0.0182
	SD	0.106	0.0362	0	0.108	0.031	0	0.028	0.022	0.0283

KUS: Kyoto University Substance, SD: standard deviation, Pre: immediately before intravitreal injection (= 24 h after latest intravitreal injection)

**Table 5:** Pharmacokinetic parameters of KUS121 after intravitreal injections.

		Day 1			Day 2			Day 3			Days 1–3		
		C_max_	T_max_	AUC_0–24 h_	C_max_	T_max_	AUC_24–48 h_	C_max_	T_max_	AUC_48–72 h_	C_max_	T_max_	AUC_0–72 h_
		(ng/mL)	(h)	(ng·h/mL)	(ng/mL)	(h)	(ng·h/mL)	(ng/mL)	(h)	(ng·h/mL)	(ng/mL)	(h)	(ng·h/mL)
Low-dose group	Average	0.0721	2.00	0.520	0.0665	2.00	0.541	0.0727	3.33	0.892	0.0849	18.7	1.96
	SD	0.0120	0.00	0.326	0.0046	0.00	0.362	0.0226	1.15	0.319	0.0132	28.9	0.90
High-dose group	Average	0.166	2.00	1.03	0.221	2.00	1.81	0.147	2.00	1.65	0.272	26.0	4.48
	SD	0.106	0.00	0.52	0.108	0.00	0.55	0.028	0.00	0.48	0.090	21.5	0.89

KUS: Kyoto University Substance, C_max_: maximum drug concentration, T_max_: time of occurrence of C_max_, AUC: area under the concentration-time curve, SD: standard deviation

Pharmacokinetics parameters were calculated by non-compartmental method.

**Fig 2:**
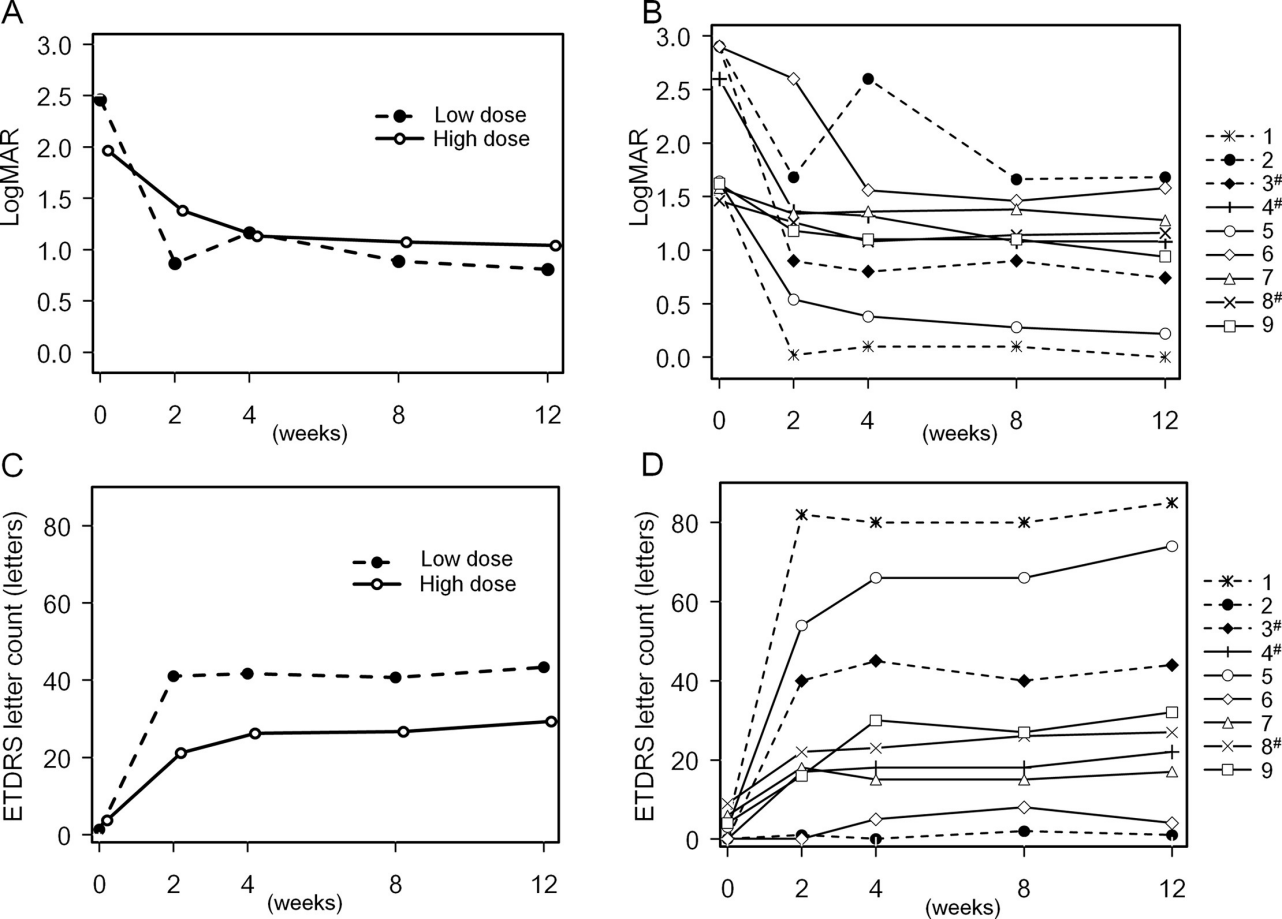
Time course of visual acuity. (A) Time course of the average logMAR tested on the ETDRS chart. (B) Time course of logMAR tested on the ETDRS chart for the nine patients enrolled in the trial. (C) Time course of the average readable ETDRS letter counts. (D) Time course of the readable ETDRS letter counts for the nine patients enrolled in the trial. Dotted line: low-dose group, full line: high-dose group. #: patient with patent cilioretinal artery in the objective eye, logMAR: logarithm of the minimum angle of resolution, ETDRS: Early Treatment Diabetic Retinopathy Study.

BCVA, measured using the ETDRS chart, improved during the observation period (Table 6 and Fig 2); logMAR BCVA at week 12 was improved (> 0.3) compared with that at baseline in all nine patients. The change in logMAR BCVA from baseline to week 12 was -1.65 ± 0.47 for the low-dose group and -0.92 ± 0.57 for the high-dose group (Table 6 and Fig 2A and 2B). The number of readable ETDRS letters at week 12 were also increased compared with that at baseline (Table 6 and Fig 2C and 2D). The change in ETDRS letter counts (week 12 vs. baseline) was 42.0 ± 40.0 letters in the low-dose group and 25.7 ± 23.7 letters in the high-dose group.

**Table 6:** Secondary outcomes related to visual functions.

	Low-dose group (n = 3)				High-dose group (n = 6)			
	Average	SD	(minimum, maximum)	95% CI	Average	SD	(minimum, maximum)	95% CI
**BCVA (ETDRS, logMAR)**								
Baseline	2.46	0.76	(1.58, 2.90)	0.57, 4.35	1.97	0.62	(1.46, 2.90)	1.32, 2.61
Week 2	0.87	0.83	(0.02, 1.68)	-1.20, 2.93	1.38	0.67	(0.54, 2.60)	0.68, 2.08
Week 4	1.17	1.29	(0.10, 2.60)	-2.04, 4.37	1.13	0.41	(0.38, 1.56)	0.70, 1.56
Week 8	0.89	0.78	(0.10, 1.66)	-1.05, 2.83	1.07	0.42	(0.28, 1.46)	0.63, 1.51
Week 12	0.81	0.84	(0.00, 1.68)	-1.29, 2.90	1.04	0.46	(0.22, 1.58)	0.56, 1.52
Baseline vs. week 12	-1.65	0.47	(-2.16, -1.22)	-2.83, -0.48	-0.92	0.57	(-1.52, -0.30)	-1.52, -0.33
**ETDRS (number of letters)**								
Baseline	1.3	2.3	(0, 4)	-4.4, 7.1	3.7	3.5	(0, 9)	0.0, 7.3
Week 2	41.0	40.5	(1, 82)	-59.6, 141.6	21.2	17.8	(0, 54)	2.5, 39.8
Week 4	41.7	40.1	(0, 80)	-58.0, 141.3	26.2	21.2	(5, 66)	3.9, 48.4
Week 8	40.7	39.0	(2, 80)	-56.2, 137.6	26.7	20.5	(8, 66)	5.1, 48.2
Week 12	43.3	42.0	(1, 85)	-61.0, 147.7	29.3	23.9	(4, 74)	4.3, 54.4
Baseline vs. week 12	42.0	40.0	(1, 81)	-57.5, 141.5	25.7	23.7	(4, 71)	0.8, 50.6
**Visual field (area of V4e) (cm**^**2**^**)**								
Baseline	36.7	14.9	(20.7, 50.3)	-0.4, 73.8	76.0	75.8	(0.0, 203.4)	-3.5, 155.6
Week 12	105.1	71.0	(29.6, 170.6)	-71.3, 281.6	90.3	70.6	(2.3, 202.8)	16.2, 164.3
Baseline vs. week 12	68.5	61.4	(8.9, 131.6)	-84.1, 221.1	14.3	11.9	(-0.6, 31.8)	1.8, 26.8
**Visual field (area of I4e) (cm**^**2**^**)**								
Baseline	5.9	7.5	(0.0, 14.4)	-12.8, 24.6	12.2	25.5	(0.0, 63.7)	-14.6, 38.9
Week 12	27.0	24.0	(0.3, 46.9)	-32.7, 86.8	29.8	37.1	(0.0, 98.6)	-9.1, 68.8
Baseline vs. week 12	21.1	18.1	(0.3, 32.5)	-23.7, 66.0	17.7	18.5	(0.0, 40.1)	-1.8, 37.1
**Visual field (VFS) (%)**								
Baseline	17.0	10.1	(8, 28)	-8.2, 42.2	24.8	20.3	(0, 45)	3.6, 46.1
Week 12	47.0	34.0	(8, 70)	-37.4, 131.4	38.7	30.3	(4, 89)	6.9, 70.5
Baseline vs. week 12	30.0	27.8	(0, 55)	-39.2, 99.2	13.8	15.3	(3, 44)	-2.2, 29.9
**Visual field (EDS) (%)**								
Baseline	28.7	16.0	(12, 44)	-11.2, 68.5	42.7	35.3	(0, 87)	5.6, 79.8
Week 12	62.7	36.1	(22, 91)	-27.0, 152.4	57.0	34.3	(5, 99)	21.0, 93.0
Baseline vs. Week 12	34.0	25.6	(10, 61)	-29.7, 97.7	14.3	9.4	(5, 31)	4.4, 24.2
**Retinal sensitivity (central 8 points) (dB)**								
Baseline	0.00	0.00	(0.00, 0.00)	-, -	1.95*	2.68	(0.00, 5.25)	-1.38, 5.28
Week 12	5.58	4.85	(0.00, 8.75)	-6.46, 17.63	5.46	6.57	(0.00, 16.25)	-1.43, 12.35
Baseline vs. week 12	5.58	4.85	(0.00, 8.75)	-6.46, 17.63	3.80*	4.94	(0.00, 11.75)	-2.33, 9.93
**Retinal sensitivity (middle 8 points) (dB)**								
Baseline	1.42	2.45	(0.00, 4.25)	-4.68, 7.51	3.95*	3.78	(0.75, 8.75)	-0.75, 8.65
Week 12	10.25	9.59	(0.00, 19.00)	-13.57, 34.07	10.45*	7.33	(3.75, 22.50)	1.35, 19.55
Baseline vs. week 12	8.83	7.80	(0.00, 14.75)	-10.53, 28.20	6.50*	5.30	(1.75, 13.75)	-0.08, 13.08
**Retinal sensitivity (peripheral 8 points) (dB)**								
Baseline	2.83	4.91	(0.00, 8.50)	-9.36, 15.02	3.53*	2.92	(0.00, 8.00)	-0.10, 7.15
Week 12	12.05	10.88	(0.00, 21.14)	-14.97, 39.07	11.33	7.78	(4.00, 25.13)	3.16, 19.50
Baseline vs. week 12	9.21	8.07	(0.00, 15.00)	-10.82, 29.25	9.27*	5.05	(4.33, 17.13)	3.00, 15.53

BCVA: best-corrected visual acuity, ETDRS: Early Treatment Diabetic Retinopathy Study, logMAR: logarithm of the minimum angle of resolution, VFS: Visual field score, EDS: Esterman disability score.

*N = 5. 95% CI was calculated based on the t-statistic.

At week 12, the proportion of patients with decimal BCVA equal to or better than 0.02 (logMAR 1.70 equivalent) was 100% (3/3) for the low-dose group and 83.3% (5/6) for the high-dose group (89% for all). Similarly, the proportion of patients with decimal BCVA equal to or better than 0.05 (logMAR 1.30 equivalent) was 66.7% (2/3) and 83.3% (5/6), respectively, and that of patients with decimal BCVA better than 0.1 (logMAR 1.00 equivalent) was 66.7% (2/3) and 33.3% (2/6), respectively (44% overall, Table 7).

**Table 7:** Visual acuity at week 12.

	Low-dose group		High-dose group	
Decimal visual acuity	(n = 3)	95% CI	(n = 6)	95% CI
≥ 0.02	3 (100.0)	29.2, 100.0	5 (83.3%)	35.9, 99.6
≥ 0.05	2 (66.7)	9.4, 99.2	5 (83.3)	35.9, 99.6
> 0.1	2 (66.7)	9.4, 99.2	2 (33.3)	4.3, 77.7

CI: confidence interval, Data are given as numbers (percentage)

Clopper-Pearson 95% CI was calculated for the proportion of patients.

**Fig 3:**
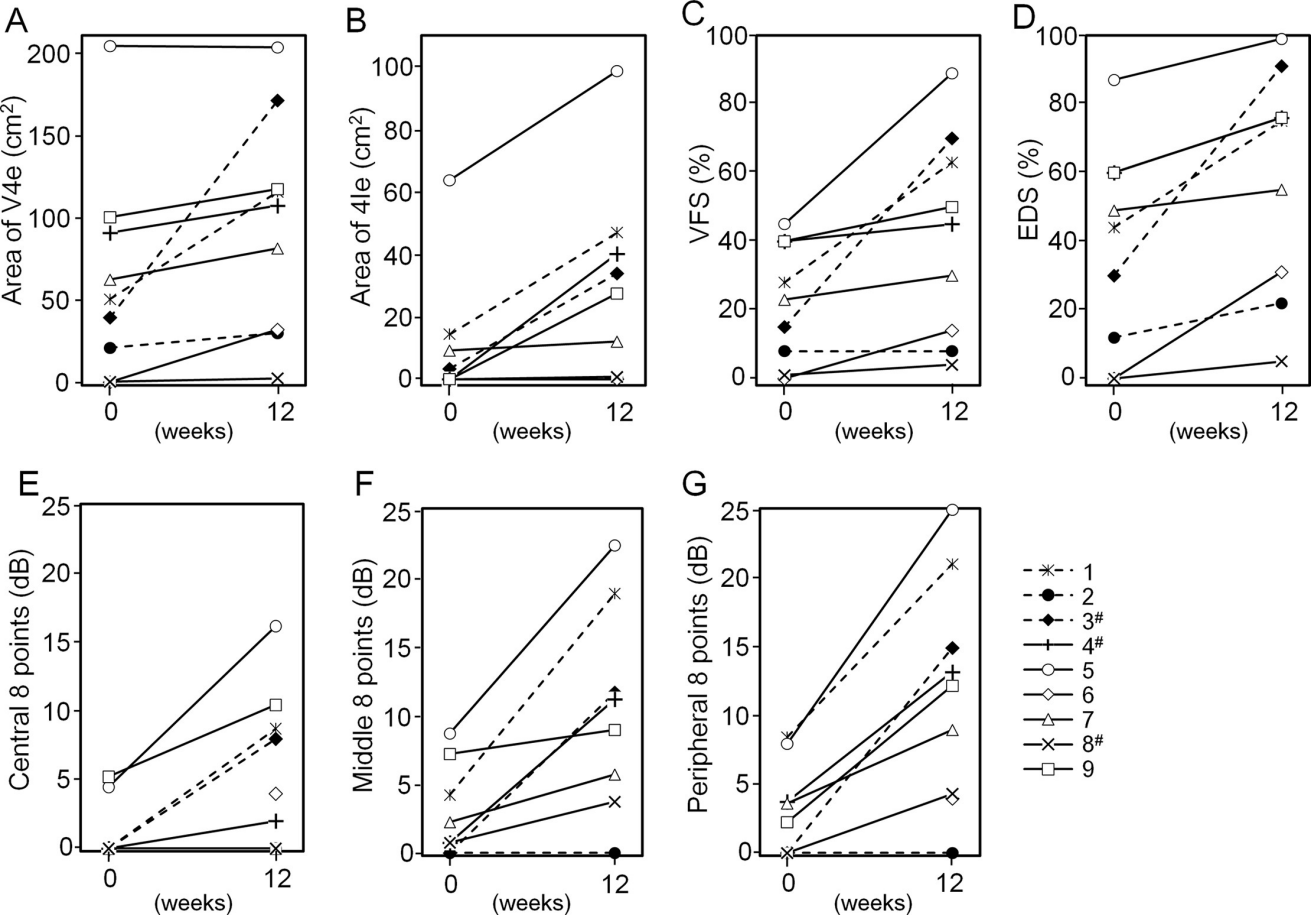
Visual field and retinal sensitivity changes from baseline to week 12 in individual patients. (A, B) Comparison of area on the visual field (V4e in A, I4e in B) (C, D) Comparison of visual field (C) and Esterman disability (D) scores on the visual field (V4e isopter). (E-G) Comparison of retinal sensitivity (central 8 points in E, middle 8 points in F, peripheral 8 points in G). #: patient with patent cilioretinal artery in the objective eye.

The patients’ visual fields, measured using Goldmann perimetry, enlarged after the treatment. The average area of V4e isopter at week 12 was 105.1 ± 71.0 cm^2^ for the low-dose group and 90.3 ± 70.6 cm^2^ for the high-dose group, which had increased from 36.7 ± 14.9 cm^2^ and 76.0 ± 75.8 cm^2^ at baseline, respectively (Table 6, Fig 3A and 3B). Visual field and Esterman disability scores, for which the normal field rates were set at 100%, were 17.0 ± 10.1% for the low-dose group and 28.7 ± 16.0% for the high-dose group at baseline, improving to 47.0 ± 34.0% and 62.7 ± 36.1% at week 12, respectively (Fig 3C and 3D). Retinal sensitivity, measured using MP-3 microperimetry, improved at 12 weeks compared with baseline in all three areas (Table 6, Fig 3E–3G).

A number of additional variables affecting visual function are summarized in S1–S5 Tables. These include the presence or absence of a patent cilioretinal artery, whether the pretreatment was received, and treatment with anti-platelet or anti-hypertensive medications prior to inclusion in the study.

## Discussion

This study reports the first-in-humans clinical trial (phase 1/2) of KUS121, a recently developed VCP modulator, for patients with CRAO, and demonstrates that intravitreal injection of KUS121 is not only safe but also effective in improving visual function.

As an ocular adverse event, an increase in intraocular pressure immediately after intravitreal injection was observed in all nine patients. Usage of a large volume (100 μL) of KUS121 solution, twice as much as typically administered during intravitreal injection of other drugs, is a likely cause of the intraocular pressure elevation. For patients with CRAO, even transient intraocular pressure elevation is considered undesirable, because it would deteriorate retinal blood flow and increase the risk of irreversible retinal dysfunction. Therefore, in this trial, we carefully measured intraocular pressure immediately after the injection of KUS121, and performed paracentesis if the intraocular pressure was exceeded the pressure before the injection by ≥ 5 mmHg. Except for immediately after the injection, intraocular pressure remained in the normal range. Thus, we considered that the intraocular pressure elevation is not a side effect of the drug, but rather an adverse event related to the intravitreal injection procedure itself. The finding that all eyes examined in this study exhibited recovery of visual function implies that such a short-term increase in intraocular pressure (for 2–3 min) would not influence the visual function. Conjunctival hemorrhage and corneal epithelium damage were also judged to be adverse events caused by the injection through the conjunctiva and lid opening during the procedure, respectively. Inflammation was not observed in either the anterior or posterior segments after injection of KUS121. Iris neovascularization, which disappeared after pan-retinal photocoagulation (laser treatment), was observed in one patient (Table 3). Ocular neovascularization has been reported to occur in 2.5–31.6% patients with CRAO [27]. Therefore, the iris neovascularization observed in this trial may be part of the disease course. However, there is the possibility that after KUS121 treatment the retina might be functional enough to generate high levels of VEGF, or that KUS121 increases sensitivity to VEGF. Worsening of retinal ischemia due to re-occlusion of the CRA is occasionally observed in the course of CRAO. Foveal structural changes due to severe inner retinal edema were observed in three patients. These phenomena might be common features of CRAO [28, 29]. Therefore, we believe that none of the adverse events are related to the administered KUS121, and we aim to confirm this in future trials.

Data on plasma concentrations of KUS121 indicated that most of the substance injected into the vitreous disappeared from the eye cavity within 24 h (Table 4). It is essential for an adequate amount of the neuroprotective agent to be constantly in contact with the retina for a number of days. For this reason, we administered KUS121 three times. This implies that KUS121 would have persisted in the vitreous for at least 4–5 days after the onset of CRAO. A more prolonged persistence of KUS121 in the eye would likely result in a more effective visual recovery. The very low maximum plasma concentration of KUS121, indicating exposure of the entire body to the substance, explains the absence of any systemic adverse effects.

Visual prognosis of CRAO has been reported to be extremely poor, regardless of treatment [30]. The natural history of the condition has been reported by Hayreh et al., based on a large case series of patients with non-arteritic CRAO [23], and by Schrag et al., based on a meta-analysis of visual outcomes of treatment for CRAO [30]. In the former report, 36% (34/95) of the patients showed improvement of visual acuity [23]. In contrast, all nine patients (100%) in this trial showed improvement of visual acuity (> 0.3 logMAR). Even in the patients with re-occlusion of the CRA, iris neovascularization treated using laser surgery or foveal detachment showed improvement of visual acuity. These results suggest that KUS121 suppresses the death of most, if not all, of the inner retinal neurons, and the functions of the surviving neurons are restored by the improved retinal blood flow or by diffusion of the choroidal blood flow. The proportion of the patients whose decimal BCVA was better than 0.1 was reported to be 18% (27/151) [23] and 18% (70/396), respectively [30]. In contrast, 44% (4/9) of the patients in this trial showed decimal BCVA better than 0.1 on their last visit (at week 12, Table 7). Although the proportion of non-blindness (decimal BCVA equal to or better than 0.05) [24] was reported to be 21% (31/151) in the study by Hayreh and Zimmerman [23], 78% (7/9) of the patients in this current trial showed values equal to or better than 0.05 (Table 7). These values apparently exceed those obtained from natural history or the results of previous studies, although we could not strictly compare the results of this trial with those of previous studies due to differences in inclusion and exclusion criteria, and follow-up duration. We further tried to compare the visual outcomes in our trial to those of the control arm of a reported randomized trial, the European Assessment Group for Lysis in the Eye Study (EAGLE) [7]. In the study, 84 CRAO patients with symptoms for 20 hours or less and BCVA < 0.5 logMAR were randomized to the control and intra-arterial fibrinolysis groups. In the control group (n = 40), conservative standard treatments such as isovolemic hemodilution, ocular massage, and administration of IOP lowering medications were performed. Furthermore, the inclusion and exclusion criteria were similar to those in our study. The average baseline BCVA of the KUS121 group in our trial was almost similar to that of the control group (p = 0.914, two-sided t test, S6 Table). In contrast, at week 4, when final visual assessments were done in the EAGLE study, the average BCVA of the KUS121 group was better than that of the control group (p = 0.031, two-sided t test, S6 Table). The average improvement of BCVA between baseline and week 4 in the KUS121 group was greater than that of the control group (p = 0.013, two-sided t test, S6 Table). In addition, visual field area, visual field scores, and retinal sensitivities improved in the present study (Table 6). These results clearly suspected the effectiveness of intravitreal injection of KUS121 in patients with acute CRAO.

Recovery of visual function after KUS121 injection may be influenced by amelioration of retinal blood flow within a week. The data shown in the S1 and S2 Tables suggest that the presence of a patent cilioretinal artery did not influence the effectiveness of KUS121, probably because only the patients with visual acuity equal to or less than 0.1 were recruited into this trial. In patients with poor visual acuity, the cilioretinal artery did not perfuse a sufficiently large area of the macula. Likewise, pre-treatments for CRAO undertaken before enrollment of the patients in this clinical trial were not found to be likely to influence the effectiveness of KUS121 (S3 Table). Other factors, e.g., age, time from CRAO onset to first injection, presence of a patent cilioretinal artery, and anti-platelet or hypertensive medications, did not appear to influence the effectiveness of KUS121 treatment in this small-scale trial.

In the inner retina, the secondary and tertiary neurons (bipolar and ganglion cells, respectively) process or transmit signals from the primary neurons, the photoreceptors. In CRAO, insufficient perfusion of the inner retina completely degrades signal phototransduction. Within several days after CRAO onset, the neuronal cells in the inner retina are thought to die due to intracellular ATP depletion and ER stress under retinal ischemia [14]. Loss of the inner retinal neurons causes irreversible visual impairment.

For more than 10 years, we have been developing KUSs as inhibitors of VCP ATPase activity and have confirmed that these substances, including KUS121, markedly suppress the decrease in intracellular ATP levels, and consequently suppress ER stress and cell death [15]. When administered to patients with acute-phase CRAO, ideally within 48 h, when retinal neurons are still surviving, KUS121 could suppress ATP depletion and ER stress, and could subsequently inhibit cell death in the retina; in most patients with CRAO, the retinal circulatory status is ameliorated to some extent across several days (S7 Table). In fact, in the present study, the intraretinal transit time improved in six (78%) patients during the first 6 days. The surviving retinal neurons are then able to recover their function due to amelioration of the retinal blood flow. In other words, via KUS121-mediated suppression of cell death by maintenance of ATP levels, a novel mechanism of neuroprotection, it is possible to improve visual function in patients with CRAO. It is believed that the retina is irreversibly damaged due to CRAO lasting for more than 4 h. However, as expected, in our clinical trial, we observed that visual restoration was feasible in these patients, even if treatment with the “neuroprotectant” was started 4 h after the onset of CRAO.

The limitation of this study was that this trial did not include a control group, and was conducted in a small sample size, due to the difficulty in including sufficient numbers of patients with CRAO exhibiting symptoms lasting less than 48 h. Given the invasiveness of the procedure, there are challenges associate with the ethics of conducting a placebo-controlled trial. In addition, the inclusion of concurrent controls would significantly increase the time required to recruit a sufficient sample size. In future trials, optimal doses will need to be reexamined in a larger patient cohort in order to confirm the safety and effectiveness of KUS121.

In conclusion, this first-in-humans clinical trial provides evidence supporting the safety of KUS121, an ATPase inhibitor of VCP. The neuroprotective potential of KUS121, based on the novel mechanism of suppressing ATP depletion, may be a viable option for first-line treatment of patients with not only CRAO, but also neurodegenerative diseases, including other ocular diseases.

## Supporting information

S1 TREND checklist TREND statement checklist. (PDF)

S1 Table Secondary outcomes related to visual functions in the patients with or without a patent cilioretinal artery. (PDF)

S2 Table Visual acuity at week 12 in the patients with or without a patent cilioretinal artery. (PDF)

S3 Table Secondary outcomes related to visual functions of patients with or without pre-treatments. (PDF)

S4 Table Secondary outcomes related to visual functions of patients with or without anti-platelet medications. (PDF)

S5 Table Secondary outcomes related to visual functions of patients with or without anti-hypertensive medications. (PDF)

S6 Table Comparison of visual outcomes. (PDF)

S7 Table Intraretinal transit time at day 6, as measured using fluorescein angiography. (PDF)

S1 Appendix A data set of this clinical trial. (XLSX)

S1 Protocol (PDF)
