# Supplementary material for: Safety and effectiveness of a novel neuroprotectant, KUS121, in patients with non-arteritic central retinal artery occlusion: An open-label, non-randomized, first-in-humans, phase 1/2 trial
Source: PLoS One. 2020 Feb 13;15(2):e0229068. doi: 10.1371/journal.pone.0229068 (PMC7018138; doi:10.1371/journal.pone.0229068)
Supplement: S1 Table — (PDF) [file pone.0229068.s002.pdf]

S1 Table. Secondary outcomes related to visual functions in the patients with or without a patent cilioretinal artery.

	Average	SD	(minimum, maximum)	95% CI	Average	SD	(minimum, maximum)	95% CI
BCVA (ETDRS, logMAR)								
Absence of a patent cilioretinal artery								
	Low-dose group (*n* = 2)				High-dose group (*n* = 4)			
Baseline	2.24	0.93	(1.58, 2.90)	-6.15, 10.63	1.94	0.64	(1.58, 2.90)	0.91, 2.96
Week 2	0.85	1.17	(0.02, 1.68)	-9.70, 11.40	1.42	0.86	(0.54, 2.60)	0.04, 2.79
Week 4	1.35	1.77	(0.10, 2.60)	-14.53, 17.23	1.10	0.52	(0.38, 1.56)	0.28, 1.92
Week 8	0.88	1.10	(0.10, 1.66)	-9.03, 10.79	1.06	0.54	(0.28, 1.46)	0.20, 1.91
Week 12	0.84	1.19	(0.00, 1.68)	-9.83, 11.51	1.01	0.59	(0.22, 1.58)	0.07, 1.94
Baseline vs. week 12	-1.40	0.26	(-1.58, -1.22)	-3.69, 0.89	-0.93	0.53	(-1.42, -0.30)	-1.78, -0.08
Presence of a patent cilioretinal artery								
	Low-dose group (*n* = 1)				High-dose group (*n* = 2)			
Baseline	2.90	-	(2.90, 2.90)	-	2.03	0.81	(1.46, 2.60)	-5.21, 9.27
Week 2	0.90	-	(0.90, 0.90)	-	1.31	0.07	(1.26, 1.36)	0.68, 1.95
Week 4	0.80	-	(0.80, 0.80)	-	1.20	0.17	(1.08, 1.32)	-0.33, 2.73
Week 8	0.90	-	(0.90, 0.90)	-	1.11	0.04	(1.08, 1.14)	0.73, 1.49
Week 12	0.74	-	(0.74, 0.74)	-	1.12	0.06	(1.08, 1.16)	0.61, 1.63
Baseline vs. week 12	-2.16	-	(-2.16, -2.16)	-	-0.91	0.86	(-1.52, -0.30)	-8.66, 6.84

BCVA (ETDRS, number of letters)								
Without a patent cilioretinal artery								
	Low-dose group (n = 2)				High-dose group (n = 4)			
Baseline	2.0	2.8	(0, 4)	-23.4, 27.4	3.3	2.5	(0, 6)	-0.7, 7.2
Week 2	41.5	57.3	(1, 82)	-473.1, 556.1	22.0	22.8	(0, 54)	-14.3, 58.3
Week 4	40.0	56.6	(0, 80)	-468.2, 548.2	29.0	26.7	(5, 66)	-13.5, 71.5
Week 8	41.0	55.2	(2, 80)	-454.5, 536.5	29.0	25.9	(8, 66)	-12.2, 70.2
Week 12	43.0	59.4	(1, 85)	-490.7, 576.7	31.8	30.4	(4, 74)	-16.6, 80.1
Baseline vs. week 12	41.0	56.6	(1, 81)	-467.2, 549.2	28.5	30.1	(4, 71)	-19.4, 76.4
With a patent cilioretinal artery								
	Low-dose group (n = 1)				High-dose group (n = 2)			
Baseline	0.0	-	(0, 0)	-	4.5	6.4	(0, 9)	-52.7, 61.7
Week 2	40.0	-	(40, 40)	-	19.5	3.5	(17, 22)	-12.3, 51.3
Week 4	45.0	-	(45, 45)	-	20.5	3.5	(18, 23)	-11.3, 52.3
Week 8	40.0	-	(40, 40)	-	22.0	5.7	(18, 26)	-28.8, 72.8
Week 12	44.0	-	(44, 44)	-	24.5	3.5	(22, 27)	-7.3, 56.3
Baseline vs. week 12	44.0	-	(44, 44)	-	20.0	2.8	(18, 22)	-5.4, 45.4

Visual field (area of V4e) (cm^2)								
Without a patent cilioretinal artery								
	Low-dose group (*n* = 2)				High-dose group (*n* = 4)			
Baseline	35.5	20.9	(20.7, 50.3)	-152.6, 223.6	91.4	85.3	(0.0, 203.4)	-44.4, 227.1
Week 12	72.4	60.5	(29.6, 115.2)	-471.4, 616.2	108.1	72.1	(31.8, 202.8)	-6.7, 222.8
Baseline vs. week 12	36.9	39.6	(8.9, 64.9)	-318.9, 392.8	16.7	13.3	(-0.6, 31.8)	-4.5, 37.9
With a patent cilioretinal artery								
	Low-dose group (*n* = 1)				High-dose group (*n* = 2)			
Baseline	39.0	-	(39.0, 39.0)	-	45.4	63.9	(0.2, 90.5)	-528.3, 619.0
Week 12	170.6	-	(170.6, 170.6)	-	54.7	74.0	(2.3, 107.0)	-610.5, 719.8
Baseline vs. week 12	131.6	-	(131.6, 131.6)	-	9.3	10.2	(2.1, 16.5)	-82.2, 100.8
Visual field (area of I4e) (cm^2)								
Without a patent cilioretinal artery								
	Low-dose group (*n* = 2)				High-dose group (*n* = 4)			
Baseline	7.2	10.2	(0.0, 14.4)	-84.3, 98.7	18.2	30.6	(0.0, 63.7)	-30.5, 67.0
Week 12	23.6	33.0	(0.3, 46.9)	-272.5, 319.7	34.5	44.2	(0.0, 98.6)	-35.8, 104.8
Baseline vs. week 12	16.4	22.8	(0.3, 32.5)	-188.2, 221.0	16.3	17.5	(0.0, 34.9)	-11.6, 44.2
With a patent cilioretinal artery								
	Low-dose group (*n* = 1)				High-dose group (*n* = 2)			
Baseline	3.3	-	(3.3, 3.3)	-	0.0	0.0	(0.0, 0.0)	-
Week 12	33.9	-	(33.9, 33.9)	-	20.5	27.8	(0.8, 40.1)	-229.2, 270.1
Baseline vs. week 12	30.6	-	(30.6, 30.6)	-	20.5	27.8	(0.8, 40.1)	-229.2, 270.1

Visual field (VFS) (%)								
	Without a patent cilioretinal artery							
	Low-dose group (*n* = 2)				High-dose group (*n* = 4)			
Baseline	18.0	14.1	(8, 28)	-109.1, 145.1	27.0	20.3	(0, 45)	-5.3, 59.3
Week 12	35.5	38.9	(8, 63)	-313.9, 384.9	45.8	32.4	(14, 89)	-5.8, 97.3
Baseline vs. week 12	17.5	24.7	(0, 35)	-204.9, 239.9	18.8	17.1	(7, 44)	-8.4, 45.9
	With a patent cilioretinal artery							
	Low-dose group (*n* = 1)				High-dose group (*n* = 2)			
Baseline	15.0	-	(15, 15)	- , -	20.5	27.6	(1, 40)	-227.3, 268.3
Week 12	70.0	-	(70, 70)	- , -	24.5	29.0	(4, 45)	-236.0, 285.0
Baseline vs. week 12	55.0	-	(55, 55)	- , -	4.0	1.4	(3, 5)	-8.7, 16.7
Visual field (EDS) (%)								
	Without a patent cilioretinal artery							
	Low-dose group (*n* = 2)				High-dose group (*n* = 4)			
Baseline	28.0	22.6	(12, 44)	-175.3, 231.3	49.0	36.4	(0, 87)	-8.9, 106.9
Week 12	48.5	37.5	(22, 75)	-288.2, 385.2	65.3	29.1	(31, 99)	19.0, 111.5
Baseline vs. week 12	20.5	14.8	(10, 31)	-112.9, 153.9	16.3	10.7	(6, 31)	-0.7, 33.2
	With a patent cilioretinal artery							
	Low-dose group (*n* = 1)				High-dose group (*n* = 2)			
Baseline	30.0	-	(30, 30)	-	30.0	42.4	(0, 60)	-351.2, 411.2
Week 12	91.0	-	(91, 91)	-	40.5	50.2	(5, 76)	-410.6, 491.6
Baseline vs. week 12	61.0	-	(61, 61)	-	10.5	7.8	(5, 16)	-59.4, 80.4

Retinal sensitivity (central 8 points) (dB)								
Without a patent cilioretinal artery								
	Low-dose group (n = 2)				High-dose group (n = 4)			
Baseline	0.00	0.00	(0.00, 0.00)	-	3.25[A]	2.84	(0.00, 5.25)	-3.80, 10.30
Week 12	4.38	6.19	(0.00, 8.75)	-51.21, 59.96	7.69	7.16	(0.00, 16.25)	-3.71, 19.09
Baseline vs. week 12	4.38	6.19	(0.00, 8.75)	-51.21, 59.96	5.67[A]	5.89	(0.00, 11.75)	-8.96, 20.29
With a patent cilioretinal artery								
	Low-dose group (n = 1)				High-dose group (n = 2)			
Baseline	0.00	-	(0.00, 0.00)	-	0.00	0.00	(0.00, 0.00)	-
Week 12	8.00	-	(8.00, 8.00)	-	1.00	1.41	(0.00, 2.00)	-11.71, 13.71
Baseline vs. week 12	8.00	-	(8.00, 8.00)	-	1.00	1.41	(0.00, 2.00)	-11.71, 13.71
Retinal sensitivity (middle 8 points) (dB)								
Without a patent cilioretinal artery								
	Low-dose group (n = 2)				High-dose group (n = 3)			
Baseline	2.13	3.01	(0.00, 4.25)	-24.88, 29.13	6.08	3.40	(2.25, 8.75)	-2.37, 14.54
Week 12	9.50	13.44	(0.00, 19.00)	-111.21, 130.21	12.42	8.88	(5.75, 22.50)	-9.65, 34.48
Baseline vs. week 12	7.38	10.43	(0.00, 14.75)	-86.33, 101.08	6.33	6.48	(1.75, 13.75)	-9.77, 22.44
With a patent cilioretinal artery								
	Low-dose group (n = 1)				High-dose group (n = 2)			
Baseline	0.00	-	(0.00, 0.00)	-	0.75	0.00	(0.75, 0.75)	- , -
Week 12	11.75	-	(11.75, 11.75)	-	7.50	5.30	(3.75, 11.25)	-40.15, 55.15
Baseline vs. week 12	11.75	-	(11.75, 11.75)	-	6.75	5.30	(3.00, 10.50)	-40.90, 54.40

Retinal sensitivity (peripheral 8 points) (dB)								
	Without patent cilioretinal artery							
	Low-dose group (n = 2)				High-dose group (n = 4)			
Baseline	4.25	6.01	(0.00, 8.50)	-49.75, 58.25	4.63[A]	3.00	(2.25, 8.00)	-2.83, 12.08
Week 12	10.57	14.95	(0.00, 21.14)	-123.75, 144.89	12.59	9.02	(4.00, 25.13)	-1.75, 26.94
Baseline vs. week 12	6.32	8.94	(0.00, 12.64)	-74.00, 86.64	10.83[A]	5.92	(5.38, 17.13)	-3.87, 25.54
	With a patent cilioretinal artery							
	Low-dose group (n = 1)				High-dose group (n = 2)			
Baseline	0.00	-	(0.00, 0.00)	-	1.88	2.65	(0.00, 3.75)	-21.95, 25.70
Week 12	15.00	-	(15.00, 15.00)	-	8.79	6.31	(4.33, 13.25)	-47.86, 65.44
Baseline vs. week 12	15.00	-	(15.00, 15.00)	-	6.92	3.65	(4.33, 9.50)	-25.91, 39.74

[A]n = 3

BCVA: best-corrected visual acuity, ETDRS: Early Treatment Diabetic Retinopathy Study, logMAR: logarithm of the minimum angle of resolution, VFS: visual field score, EDS: Esterman disability score. 95% CI was calculated based on the t-statistic.
