# Supplementary material for: Safety and effectiveness of a novel neuroprotectant, KUS121, in patients with non-arteritic central retinal artery occlusion: An open-label, non-randomized, first-in-humans, phase 1/2 trial
Source: PLoS One. 2020 Feb 13;15(2):e0229068. doi: 10.1371/journal.pone.0229068 (PMC7018138; doi:10.1371/journal.pone.0229068)
Supplement: S2 Table — (PDF) [file pone.0229068.s003.pdf]

S2 Table. Visual acuity at week 12 in the patients with or without a patent cilioretinal artery.

	Without a patent cilioretinal artery			
	Low-dose group (*n* = 2)		High-dose group (*n* = 4)	
Decimal visual acuity	(%)	95% CI	(%)	95% CI
≥ 0.02	2 (100.0)	15.8, 100.0	3 (75.0)	19.4, 99.4
≥ 0.05	1 (50.0)	1.3, 98.7	3 (75.0)	19.4, 99.4
> 0.1	1 (50.0)	1.3, 98.7	1 (25.0)	0.6, 80.6
	With a patent cilioretinal artery			
	Low-dose group (*n* = 1)		High-dose group (*n* = 2)	
Decimal visual acuity	(%)	95% CI	(%)	95% CI
≥ 0.02	1 (100.0)	2.5, 100.0	2 (100.0)	15.8, 100.0
≥ 0.05	1 (100.0)	2.5, 100.0	2 (100.0)	15.8, 100.0
> 0.1	1 (100.0)	2.5, 100.0	1 (50.0)	1.3, 98.7

CI: confidence interval. Clopper-Pearson 95% CI was calculated for the proportion of patients.
