# Supplementary material for: Safety and effectiveness of a novel neuroprotectant, KUS121, in patients with non-arteritic central retinal artery occlusion: An open-label, non-randomized, first-in-humans, phase 1/2 trial
Source: PLoS One. 2020 Feb 13;15(2):e0229068. doi: 10.1371/journal.pone.0229068 (PMC7018138; doi:10.1371/journal.pone.0229068)
Supplement: S3 Table — (PDF) [file pone.0229068.s004.pdf]

S3 Table. Secondary outcomes related to visual functions of patients with or without pre-treatments.

	Average	SD	(minimum, maximum)	95% CI	Average	SD	(minimum, maximum)	95% CI
BCVA (ETDRS, logMAR)								
Without pre-treatments								
	Low-dose group (n = 1)				High-dose group (n = 2)			
Baseline	1.58	-	(1.58, 1.58)	-	2.26	0.91	(1.62, 2.90)	-5.87, 10.39
Week 2	0.02	-	(0.02, 0.02)	-	1.89	1.00	(1.18, 2.60)	-7.13, 10.91
Week 4	0.10	-	(0.10, 0.10)	-	1.33	0.33	(1.10, 1.56)	-1.59, 4.25
Week 8	0.10	-	(0.10, 0.10)	-	1.28	0.25	(1.10, 1.46)	-1.01, 3.57
Week 12	0.00	-	(0.00, 0.00)	-	1.26	0.45	(0.94, 1.58)	-2.81, 5.33
Baseline vs. week 12	-1.58	-	(-1.58, -1.58)	-	-1.00	0.45	(-1.32, -0.68)	-5.07, 3.07
With pre-treatments								
	Low-dose group (n = 2)				High-dose group (n = 4)			
Baseline	2.90	0.00	(2.90, 2.90)	-	1.82	0.53	(1.46, 2.60)	0.98, 2.66
Week 2	1.29	0.55	(0.90, 1.68)	-3.67, 6.25	1.13	0.39	(0.54, 1.36)	0.50, 1.75
Week 4	1.70	1.27	(0.80, 2.60)	-9.74, 13.14	1.04	0.45	(0.38, 1.36)	0.31, 1.76
Week 8	1.28	0.54	(0.90, 1.66)	-3.55, 6.11	0.97	0.48	(0.28, 1.38)	0.21, 1.73
Week 12	1.21	0.66	(0.74, 1.68)	-4.76, 7.18	0.94	0.48	(0.22, 1.28)	0.17, 1.71
Baseline vs. week 12	-1.69	0.67	(-2.16, -1.22)	-7.66, 4.28	-0.89	0.68	(-1.52, -0.30)	-1.96, 0.19

BCVA (ETDRS, number of letters)								
			Without pre-treatments					
	Low-dose group (n = 1)				High-dose group (n = 2)			
Baseline	4.0	-	(4, 4)	-	2.0	2.8	(0, 4)	-23.4, 27.4
Week 2	82.0	-	(82, 82)	-	8.0	11.3	(0, 16)	-93.6, 109.6
Week 4	80.0	-	(80, 80)	-	17.5	17.7	(5, 30)	-141.3, 176.3
Week 8	80.0	-	(80, 80)	-	17.5	13.4	(8, 27)	-103.2, 138.2
Week 12	85.0	-	(85, 85)	-	18.0	19.8	(4, 32)	-159.9, 195.9
Baseline vs. week 12	81.0	-	(81, 81)	-	16.0	17.0	(4, 28)	-136.5, 168.5
			With pre-treatments					
	Low-dose group (n = 2)				High-dose group (n = 4)			
Baseline	0.0	0.0	(0, 0)	-	4.5	3.9	(0, 9)	-1.7, 10.7
Week 2	20.5	27.6	(1, 40)	-227.3, 268.3	27.8	17.6	(17, 54)	-0.3, 55.8
Week 4	22.5	31.8	(0, 45)	-263.4, 308.4	30.5	23.9	(15, 66)	-7.5, 68.5
Week 8	21.0	26.9	(2, 40)	-220.4, 262.4	31.3	23.6	(15, 66)	-6.3, 68.8
Week 12	22.5	30.4	(1, 44)	-250.7, 295.7	35.0	26.3	(17, 74)	-6.9, 76.9
Baseline vs. week 12	22.5	30.4	(1, 44)	-250.7, 295.7	30.5	27.4	(11, 71)	-13.1, 74.1

BCVA: best-corrected visual acuity, ETDRS: Early Treatment Diabetic Retinopathy Study, logMAR: logarithm of the minimum angle of resolution.

95% CI was calculated based on the t-statistic.
