# Supplementary material for: Safety and effectiveness of a novel neuroprotectant, KUS121, in patients with non-arteritic central retinal artery occlusion: An open-label, non-randomized, first-in-humans, phase 1/2 trial
Source: PLoS One. 2020 Feb 13;15(2):e0229068. doi: 10.1371/journal.pone.0229068 (PMC7018138; doi:10.1371/journal.pone.0229068)
Supplement: S4 Table — (PDF) [file pone.0229068.s005.pdf]

S4 Table. Secondary outcomes related to visual functions of patients with or without anti-platelet medications.

	Average	SD	(minimum, maximum)	95% CI	Average	SD	(minimum, maximum)	95% CI
BCVA (ETDRS, logMAR)								
Without anti-platelet medications								
	Low-dose group (n = 2)				High-dose group (n = 4)			
Baseline	2.90	0.00	(2.90, 2.90)	-	1.82	0.53	(1.46, 2.60)	0.98, 2.65
Week 2	1.28	0.54	(0.90, 1.66)	-3.55, 6.11	1.18	0.14	(1.08, 1.38)	0.95, 1.40
Week 4	1.21	0.66	(0.74, 1.68)	-4.76, 7.18	1.12	0.14	(0.94, 1.28)	0.89, 1.34
Week 8	1.29	0.55	(0.90, 1.68)	-3.67, 6.25	1.29	0.08	(1.18, 1.36)	1.15, 1.42
Week 12	1.70	1.27	(0.80, 2.60)	-9.74, 13.14	1.22	0.15	(1.08, 1.36)	0.98, 1.45
Baseline vs. week 12	-1.69	0.66	(-2.16, -1.22)	-7.66, 4.28	-0.70	0.58	(-1.52, -0.30)	-1.62, 0.22
With anti-platelet medications								
	Low-dose group (n = 1)				High-dose group (n = 2)			
Baseline	1.58	-	(1.58, 1.58)	-	2.27	0.89	(1.64, 2.90)	-5.73, 10.27
Week 2	0.10	-	(0.10, 0.10)	-	0.87	0.83	(0.28, 1.46)	-6.63, 8.37
Week 4	0.00	-	(0.00, 0.00)	-	0.90	0.96	(0.22, 1.58)	-7.74, 9.54
Week 8	0.02	-	(0.02, 0.02)	-	1.57	1.46	(0.54, 2.60)	-11.52, 14.66
Week 12	0.10	-	(0.10, 0.10)	-	0.97	0.83	(0.38, 1.56)	-6.53, 8.47
Baseline vs. week 12	-1.58	-	(-1.58, -1.58)	-	-1.37	0.07	(-1.42, -1.32)	-2.01, -0.73

BCVA (ETDRS, number of letters)								
Without anti-platelet medications								
	Low-dose group (n = 2)				High-dose group (n = 4)			
Baseline	0.0	0.0	(0, 0)	-	4.8	3.8	(0, 9)	-1.3, 10.8
Week 2	21.0	26.9	(2, 40)	-220.4, 262.4	21.5	5.9	(15, 27)	12.1, 30.9
Week 4	22.5	30.4	(1, 44)	-250.7, 295.7	24.5	6.5	(17, 32)	14.2, 34.8
Week 8	20.5	27.6	(1, 40)	-227.3, 268.3	18.3	2.6	(16, 22)	14.1, 22.4
Week 12	22.5	31.8	(0, 45)	-263.4, 308.4	21.5	6.6	(15, 30)	11.1, 31.9
Baseline vs. week 12	22.5	30.4	(1, 44)	-250.7, 295.7	19.8	7.1	(11, 28)	8.4, 31.1
With anti-platelet medications								
	Low-dose group (n =1)				High-dose group (n =2)			
Baseline	4.0	-	(4, 4)	-	1.5	2.1	(0, 3)	-17.6, 20.6
Week 2	80.0	-	(80, 80)	-	37.0	41.0	(8, 66)	-331.5, 405.5
Week 4	85.0	-	(85, 85)	-	39.0	49.5	(4, 74)	-405.7, 483.7
Week 8	82.0	-	(82, 82)	-	27.0	38.2	(0, 54)	-316.1, 370.1
Week 12	80.0	-	(80, 80)	-	35.5	43.1	(5, 66)	-352.0, 423.0
Baseline vs. week 12	81.0	-	(81, 81)	-	37.5	47.4	(4, 71)	-388.2, 463.2

BCVA: best-corrected visual acuity, ETDRS: Early Treatment Diabetic Retinopathy Study, logMAR: logarithm of the minimum angle of resolution.

95% CI was calculated based on t-statistic.
