# Supplementary material for: Safety and effectiveness of a novel neuroprotectant, KUS121, in patients with non-arteritic central retinal artery occlusion: An open-label, non-randomized, first-in-humans, phase 1/2 trial
Source: PLoS One. 2020 Feb 13;15(2):e0229068. doi: 10.1371/journal.pone.0229068 (PMC7018138; doi:10.1371/journal.pone.0229068)
Supplement: S5 Table — (PDF) [file pone.0229068.s006.pdf]

S5 Table. Secondary outcomes related to visual functions of patients with or without anti-hypertensive medications.

	Average	SD	(minimum, maximum)	95% CI	Average	SD	(minimum, maximum)	95% CI
BCVA (ETDRS, logMAR)								
Without anti-hypertensive medications								
	Low-dose group (*n* = 1)				High-dose group (*n* = 2)			
Baseline	1.58	-	(1.58, 1.58)	-	1.54	0.11	(1.46, 1.62)	0.52, 2.56
Week 2	0.10	-	(0.10, 0.10)	-	1.12	0.03	(1.10, 1.14)	0.87, 1.37
Week 4	0.00	-	(0.00, 0.00)	-	1.05	0.16	(0.94, 1.16)	-0.35, 2.45
Week 8	0.02	-	(0.02, 0.02)	-	1.22	0.06	(1.18, 1.26)	0.71, 1.73
Week 12	0.10	-	(0.10, 0.10)	-	1.09	0.01	(1.08, 1.10)	0.96, 1.22
Baseline vs. week 12	-1.58	-	(-1.58, -1.58)	-	-0.49	0.27	(-0.68, -0.30)	-2.90, 1.92
With anti-hypertensive medications								
	Low-dose group (*n* = 2)				High-dose group (*n* = 4)			
Baseline	2.90	0.00	(2.90, 2.90)	-	2.18	0.67	(1.58, 2.90)	1.11, 3.25
Week 2	1.28	0.54	(0.90, 1.66)	-3.55, 6.11	1.05	0.54	(0.28, 1.46)	0.19, 1.91
Week 4	1.21	0.66	(0.74, 1.68)	-4.76, 7.18	1.04	0.58	(0.22, 1.58)	0.11, 1.97
Week 8	1.29	0.55	(0.90, 1.68)	-3.67, 6.25	1.46	0.85	(0.54, 2.60)	0.11, 2.81
Week 12	1.70	1.27	(0.80, 2.60)	-9.74, 13.14	1.16	0.53	(0.38, 1.56)	0.32, 1.99
Baseline vs. week 12	-1.69	0.66	(-2.16, -1.22)	-7.66, 4.28	-1.14	0.57	(-1.52, -0.30)	-2.04, -0.24

BCVA (ETDRS, number of letters)								
	Without anti-hypertensive medications							
	Low-dose group (n = 1)				High-dose group (n = 2)			
Baseline	4.0	-	(4, 4)	-	6.5	3.5	(4, 9)	-25.3, 38.3
Week 2	80.0	-	(80, 80)	-	26.5	0.7	(26, 27)	20.2, 32.9
Week 4	85.0	-	(85, 85)	-	29.5	3.5	(27, 32)	-2.3, 61.3
Week 8	82.0	-	(82, 82)	-	19.0	4.2	(16, 22)	-19.1, 57.1
Week 12	80.0	-	(80, 80)	-	26.5	5.0	(23, 30)	-18.0, 71.0
Baseline vs. week 12	81.0	-	(81, 81)	-	23.0	7.1	(18, 28)	-40.5, 86.5
	With anti-hypertensive medications							
	Low-dose group (n = 2)				High-dose group (n = 4)			
Baseline	0.0	0.0	(0, 0)	-	2.3	2.9	(0, 6)	-2.3, 6.8
Week 2	21.0	26.9	(2, 40)	-220.4, 262.4	26.8	26.5	(8, 66)	-15.4, 68.9
Week 4	22.5	30.4	(1, 44)	-250.7, 295.7	29.3	30.8	(4, 74)	-19.7, 78.2
Week 8	20.5	27.6	(1, 40)	-227.3, 268.3	22.3	22.7	(0, 54)	-13.9, 58.4
Week 12	22.5	31.8	(0, 45)	-263.4, 308.4	26.0	27.2	(5, 66)	-17.3, 69.3
Baseline vs. week 12	22.5	30.4	(1, 44)	-250.7, 295.7	27.0	30.3	(4, 71)	-21.1, 75.1

BCVA: best-corrected visual acuity, ETDRS: Early Treatment Diabetic Retinopathy Study, logMAR: logarithm of the minimum angle of resolution.

95% CI was calculated based on the t-statistic.
