# Supplementary material for: Safety and effectiveness of a novel neuroprotectant, KUS121, in patients with non-arteritic central retinal artery occlusion: An open-label, non-randomized, first-in-humans, phase 1/2 trial
Source: PLoS One. 2020 Feb 13;15(2):e0229068. doi: 10.1371/journal.pone.0229068 (PMC7018138; doi:10.1371/journal.pone.0229068)
Supplement: S6 Table — (PDF) [file pone.0229068.s007.pdf]

S6 Table. Comparison of visual outcomes.

	KUS121 (n = 9)			Control group[*1] (n = 40)			p-value[*2]
**BCVA (ETDRS, logMAR)**	**Average**	**SD**	**95% CI[*3]**	**Average**	**SD**	**95% CI[*2]**	
Baseline	2.13	0.67	1.62, 2.64	2.11	0.49	1.95, 2.27	0.914
Week 4	1.14	0.72	0.59, 1.70	1.67	0.41	1.47, 1.87	0.031
Baseline vs. week 4	-0.99	0.65	-1.49, -0.49	-0.44	0.55	-0.62, -0.27	0.013

[*1] Control arm of the European Assessment Group for Lysis in the Eye Study (EAGLE). Data from Table 3 in Ophthalmology 2010;117:1367–1375 (ref. (7)).

[*2] two-sided t test

[*3] 95%CI was calculated based on the t-statistic.
