# Supplementary material for: Safety and effectiveness of a novel neuroprotectant, KUS121, in patients with non-arteritic central retinal artery occlusion: An open-label, non-randomized, first-in-humans, phase 1/2 trial
Source: PLoS One. 2020 Feb 13;15(2):e0229068. doi: 10.1371/journal.pone.0229068 (PMC7018138; doi:10.1371/journal.pone.0229068)
Supplement: S7 Table — (PDF) [file pone.0229068.s008.pdf]

S7 Table. Intraretinal transit time at day 6, as measured using fluorescein angiography.

Patient No.	Affected eye	Intraretinal transit time (s)	Improvement of intraretinal transit time compared with the baseline
Low-dose group			
1	R	35	+
2	R	120	+
3	L	42	+
High-dose group			
4	R	> 600	
5	R	6	
6	R	81	+
7	L	> 300	
8	R	21	+
9	L	37	+
