# Supplementary material for: Safety and effectiveness of a novel neuroprotectant, KUS121, in patients with non-arteritic central retinal artery occlusion: An open-label, non-randomized, first-in-humans, phase 1/2 trial
Source: PLoS One. 2020 Feb 13;15(2):e0229068. doi: 10.1371/journal.pone.0229068 (PMC7018138; doi:10.1371/journal.pone.0229068)
Supplement: S1 Protocol — (PDF) [file pone.0229068.s010.pdf]

Phase I/II Study of KUS121 for Safety and Efficacy Evaluation in Non-Arteritic Central Retinal Artery Occlusion after Intravitreal Administration for 3 Days

An Ectract from the Clinical Study Report

Sponsor investigator
Hanako Ikeda
Institute for Advancement of Clinical and Translational Science
Kyoto University Hospital

Protocol No.: iACT-15014
Date of creation of CSR: 28 September 2018
Version No.: Ver. 1

1. Title Page

Study title	Phase I/II study of KUS121 for safety and efficacy evaluation in non-arteritic central retinal artery occlusion after intravitreal administration for 3 days
Name of test drug	KUS121
Indication studied	Non-arteritic central retinal artery occlusion
Design/comparator/objectives/duration/dosage/patient population	Design: Open-label, dose-escalation, two-cohort study<br>Dose and regimen: KUS121 was intravitreally administered in the form of 250 μg/mL (low dose group) or 500 μg/mL (high dose group) solution at a volume of 100 μL once daily for 3 days. The dose of KUS121 was 25 μg/eye for the low dose and 50 μg/eye for the high dose.<br>Patient population: Patients with non-arteritic central retinal artery occlusion in the acute phase after the onset
Sponsor investigator	Hanako Ikeda, Institute for Advancement of Clinical and Translational Science, Kyoto University Hospital
Protocol number	iACT-15014
Phase of development	Phase I/II
Study initiation date	22 November 2016 (date the first subject provided informed consent)
Early study termination	Not applicable
Study completion date	2 March 2018 (date the last subject completed all observations)
Medical officer	Not included
Name of the sponsor investigator's responsible person/contact information of person in charge	Hanako Ikeda<br>Retinal Nerve Protective Therapy Project<br>Institute for Advancement of Clinical and Translational Science, Kyoto University Hospital<br>54 Shogoin-Kawahara-cho, Sakyo-ku, Kyoto 606-8507, Japan<br>Tel: 075-751-3248　Fax: 075-752-0933<br><br>Department of Clinical Innovative Medicine, Institute for Advancement of Clinical and Translational Science, Kyoto University Hospital<br>Eriko Sumi<br>54 Shogoin-Kawahara-cho, Sakyo-ku, Kyoto 606-8507, Japan<br>Tel: 075-751-4739　Fax: 075-751-4214

2. Table of Contents

1. Title Page 1
2. Table of Contents 2
3. List of Abbreviations and Definition of Terms 4
4. Investigators and Study Administrative Structure 6
5. Introduction 8
5.1 Pharmaceutical Development, Mechanism of Action, and Potential Efficacy 8
5.2 Treatment of Central Retinal Artery Occlusion 9
5.3 Expected Clinical Position of KUSs 10
6. Study Objectives 11
7. Investigational Plan 12
7.1 Overall Study Design and Plan 12
7.2 Discussion of Study Design, Including the Choice of Control Groups 13
7.3 Selection of Study Population 14
7.3.1 Inclusion Criteria 14
7.3.2 Exclusion Criteria 14
7.3.3 Removal of Subjects from Therapy or Assessment 15
7.4 Treatments 17
7.4.1 Treatments Administered 17
7.4.2 Identity of Investigational Products 19
7.4.3 Method of Assigning Subjects to Treatment Groups 22
7.4.4 Selection of Doses in the Study 23
7.4.5 Selection and Timing of Dose for Each Subject 27
7.4.6 Blinding 27
7.4.7 Prior and Concomitant Therapies 27
7.4.8 Treatment Compliance 29
7.5 Efficacy and Safety Variables 29
7.5.1 Efficacy and Safety Measurements Assessed and Flow Chart 29
7.5.2 Appropriateness of Measurements 45
7.5.3 Definitions of Endpoints 45
7.5.4 Drug Concentration Measurements 47
7.6 Data Quality Assurance 47
7.6.1 Data and Safety Monitoring Committee (DSMC) 48
7.6.2 Study Quality Control 48
7.6.3 Study Quality Assurance 48
7.7 Statistical Methods Planned in the Protocol and Determination of Sample Size 48
7.7.1 Statistical and Analytical Plans 48

7.7.2 Determination of Sample Size 52

3. List of Abbreviations and Definition of Terms

Abbreviation		
AAA	ATPases associated with diverse cellular activities	-
ATP	Adenosine triphosphate	
ATPase	ATPase	
AUC	Area under the concentration-time curve	
AUC_{0-24h}	Area under the concentration-time curve from time zero to 24 hours	
AUC_{24-48h}	Area under the concentration-time curve from time 24 to 48 hours	
AUC_{48-72h}	Area under the concentration-time curve from time 48 to 72 hours	
CI	Confidence interval	
C_{max}	Maximum concentration	
CK	Creatine Kinase	
CRP	C-reactive protein	
DSMC	Data and Safety Monitoring Committee	
EAS	Efficacy analysis set	
ECG	Electrocardiography	
ERG	Electroretinogram	
ERG	Electroretinography	
ETDRS	Early Treatment Diabetic Retinopathy Study	
EDS	Esterman disability score	
GCP	Good Clinical Practice	
GP	Goldmann Perimeter	
IC_{50}	50% inhibitory concentration	
IC_{90}	90% inhibitory concentration	
IRB	Institutional review board	
KUS	Kyoto University Substance	
LC/MS/MS	Liquid chromatograph-tandem mass spectrometer	
LDH	Lactate Dehydrogenase	
LogMAR	Logarithm of Minimum Angle of Resolution	
MA	Mean of All area	
MBR	Mean Blur Rate	

Abbreviation		
MP-3	Micro Perimeter-3	
MT	Mean of Tissue area	-
MV	Mean of Vascular area	-
NGSP	National Glycohemoglobin Standardization Program	-
NOAEL	No-observed adverse effect level	
NSF	N-ethylmaleimide-sensitive fusion protein	
SAS	Safety analysis set	
OCT	Optical coherence tomography	
PK/PD	Pharmacokinetics/Pharmacodynamics	
SD	Standard deviation	
T_{max}	Time of occurrence of C_{max}	
VCP	Valosin-containing protein	
VFS	Visual Field Score	-

4. Investigators and Study Administrative Structure

(1) Sponsor investigator (investigator)

[Duties] To be totally responsible for planning, conduct, and operational management of the study

Associate Professor, Institute for Advancement of Clinical and Translational Science, Kyoto University Hospital

Hanako Ikeda

54 Shogoin-Kawahara-cho, Sakyo-ku, Kyoto 606-8507, Japan

Tel: 075-751-3248　Fax: 075-752-0933

(2) Study site

Department of Ophthalmology, Kyoto University Hospital

(3) Plasma concentration measuring facility

[Duties] To measure plasma KUS121 concentrations

Pharmacokinetics and Bioanalysis Center, Shin Nippon Biomedical Laboratories, Ltd.

Kainan Intelligent Park, 16-1 Minami-Akasaka, Kainan, Wakayama 642-0017, Japan

Tel: 073-483-8881　Fax: 073-483-7377

(4) Data and Safety Monitoring Committee (DSMC)

[Duties] To conduct discussions and recommend actions at the request of the sponsor investigator after the start of the study when a serious adverse event occurs, any problem requires premature termination of the entire study, or the sponsor investigator asks for discussion by the DSMC for any other reason

Department of Ophthalmology, The Hospital of Hyogo College of Medicine

Osamu Mimura

Department of Ophthalmology, Kyoto Prefectural University of Medicine

Chie Sotozono

Department of Ophthalmology, Aichi Medical University

Motohiro Kamei

(5) Study manager

[Duties] To prepare for and manage the study through management of study progression, communications among individuals involved in the study, management of study-related documents, preparation of draft documented procedures, etc.

Department of Clinical Innovative Medicine, Institute for Advancement of Clinical and Translational Science, Kyoto University Hospital

Eriko Sumi

54 Shogoin-Kawahara-cho, Sakyo-ku, Kyoto 606-8507, Japan
Tel: 075-751-4739　Fax: 075-751-4214

(6) Data center

Department of Data Science, Institute for Advancement of Clinical and Translational Science, Kyoto University Hospital
54 Shogoin-Kawahara-cho, Sakyo-ku, Kyoto 606-8507, Japan
Tel: 075-751-3397　Fax: 075-751-3399

(7) Statistical analysis manager, statistician, and data management manager

[Duties] To register patients, manage data, and perform statistical analyses in the study

Statistical analysis manager

Department of Data Science, Institute for Advancement of Clinical and Translational Science, Kyoto University Hospital
Satoshi Morita

Statistician

Department of Data Science, Institute for Advancement of Clinical and Translational Science, Kyoto University Hospital
Hiroyasu Abe

Data management manager

Department of Data Science, Institute for Advancement of Clinical and Translational Science, Kyoto University Hospital
Akemi Kinoshita

(8) Monitoring manager

[Duties] To be responsible for monitoring in the study

Department of Data Science, Institute for Advancement of Clinical and Translational Science, Kyoto University Hospital
Kayoko Enomoto

(9) Auditor

[Duties] To investigate whether the study has been conducted in accordance with GCP and the protocol in order to assure the reliability of materials collected in the course of the study

Quality Assurance Division, Clinical Research and Medical Innovation Center, Hokkaido University Hospital
Kouji Nakamura

5. Introduction

5.1 Pharmaceutical Development, Mechanism of Action, and Potential Efficacy

Kyoto University Substances (KUSs) are new compounds developed by Kyoto University as inhibitors of adenosine triphosphatase (ATPase) activity of valosin-containing protein (VCP)[1)]. VCP, an ATPase belonging to the AAA (ATPases associated with diverse cellular activities) family, may play an important role in inducing cellular stress responses to accumulation of abnormal proteins or oxidative stress and is found in all body cells reportedly with various physiological activities, including proteasome-mediated protein degradation, endoplasmic reticulum-mediated protein degradation, cell cycle control, and membrane fusion[2),3)]. VCP is also found in all retinal neural cells[1)].

KUS121 and its structural analogues created during the development (KUSs: KUS69, 94, and 187) are shown to suppress ATP consumption (decrease) in cultured cells and reduce endoplasmic reticulum stress in cultured cells and ocular disease models[1),4)-7)]. Endoplasmic reticulum stress is generally considered to be induced by excessive accumulation of misfolded or denatured proteins in the endoplasmic reticulum that is detrimental to cells, and it is known that excessive and irreversibly damaging endoplasmic reticulum stress on cells activates apoptosis-inducing factors such as Bax[8)] to induce cell death[9)]. In fact, KUS121 is shown to prevent cell death *in vivo*[1)] and protect almost all retinal cells, including retinal ganglion cells, from death. In addition, KUSs not only inhibited the degeneration of retinal cells, but also maintained their function on electroretinograms in animal models of glaucoma[7)], retinal pigment degeneration[1), 4)], age-related macular degeneration[6)], and ischemic ocular disease[5)]. Since the ATPase activity of VCP inhibited by KUSs accounts for approximately 20% to nearly 40% of ATPase activity in the cytoplasmic soluble fraction[1)], prevention of cell death by KUSs may rely on prevention of diversion into mitochondrial and other cell death pathways by inhibiting the ATPase activity of VCP abundant in the cytoplasm to reverse the age-, disease-, or ischemia-related decrease in cellular ATP and reducing endoplasmic reticulum stress. Hence, KUSs are expected to be potential new neuroprotectants for refractory ocular disease. Among the KUSs, KUS121[1)] is being developed as a candidate for clinical use.

<Central retinal artery occlusion_untreated>

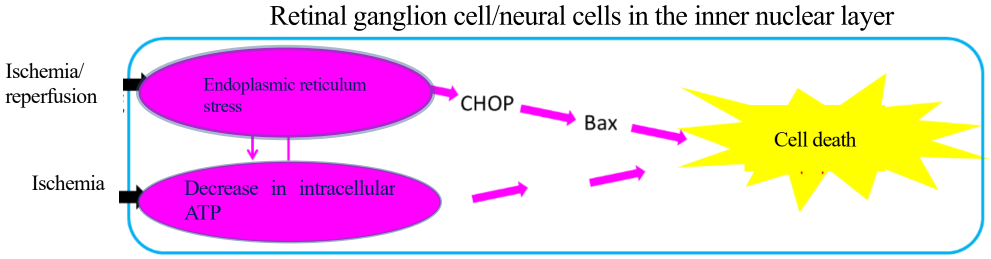

<Treatment with KUS121>

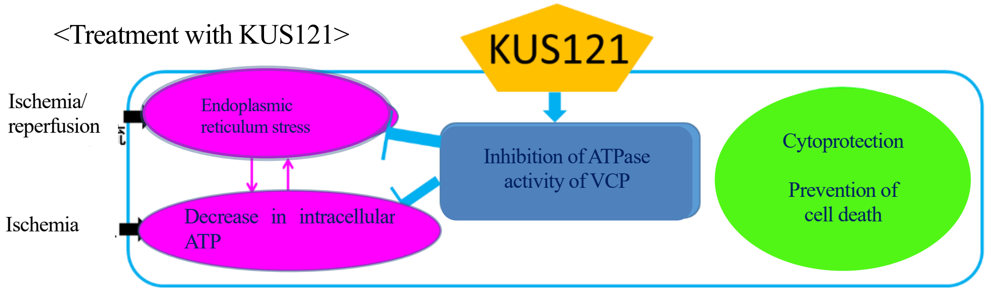

5.2 Treatment of Central Retinal Artery Occlusion

Central retinal artery occlusion is characterized by sudden severe and mostly irreversible vision/visual field loss due to occlusion of the central retinal artery nourishing the inner retinal layer (including retinal ganglion cells). Central retinal artery occlusion is classified into arteritic occlusion (associated with generalized aortitis) and non-arteritic occlusion (associated with arteriosclerotic disease) according to the cause of occlusion. In this study, only non-arteritic occlusion is addressed as central retinal artery occlusion. Ischemia-induced dysfunction of retinal ganglion cells with high oxygen consumption results in sudden vision/visual field loss, and retinal ganglion cell death, which occurs in the following few days, perpetuates the loss. Ischemic cell death is reported to be induced by cellular ATP depletion and subsequent endoplasmic reticulum stress[10)].

Central retinal artery occlusion is diagnosed based on a history of sudden unilateral vision/visual field loss, funduscopy findings, etc. No diagnostic criteria have been established in or outside Japan.

No consensus has been reached on treatment for improving visual acuity. Procedures currently taken for some patients or at some institutions are broadly classified into those intended to reopen an affected blood vessel in the acute phase (usually within 6 hours of onset) (e.g., eye massage, fibrinolytic therapy) and those intended to supply more oxygen and glucose to the retina later (e.g., intraocular pressure-lowering therapy, vasodilator therapy, hyperbaric oxygen therapy). However, these treatments have not been verified to be effective or have been reported to worsen the prognosis according to a meta-analysis[11)-13)]. In brief, since no consensus has been reached on treatment for improving the prognosis of visual acuity, development of an innovative treatment in terms of cytoprotection is awaited.

5.3 Expected Clinical Position of KUSs

Central retinal artery occlusion is characterized by severe and mostly irreversible vision/visual field loss and generally examined several hours after the onset. No effective standard therapy is available.

Since cell death is irreversible in central retinal artery occlusion, it is therapeutically important to prevent death of retinal cells damaged by ischemia or reperfusion. Vision/visual field loss may be alleviated by inhibiting cellular ATP depletion and reducing endoplasmic reticulum stress before all cells in the inner retinal layer die to prevent death of as many retinal cells as possible.

KUS121 may prevent retinal cell death by inhibiting VCP ATPase to inhibit cellular ATP depletion and reducing endoplasmic reticulum stress. Since an occluded central retinal artery is usually reopened in a few days, surviving retinal cells are expected to regain function owing to recovery of blood flow. KUS121 is being developed as first-line conservative treatment for central retinal artery occlusion, for which no treatment is currently available.

6. Study Objectives

The objective of this study was to evaluate the safety, tolerability, pharmacokinetics, and efficacy of KUS121 in non-arteritic central retinal artery occlusion after repeated intravitreal administration for 3 days.

7. Investigational Plan

7.1 Overall Study Design and Plan

This was a Phase I/II open-label study. The overall study design and plan are described below. The protocol and sample case report form are provided in Appendix 16.1.1 and Appendix 16.1.2, respectively.

(1) Treatment studied

KUS121 was intravitreally administered in the form of 250 µg/mL (low dose group) or 500 µg/mL (high dose group) solution at a volume of 100 µL once daily for 3 days. The dose of KUS121 was 25 µg/eye for the low dose and 50 µg/eye for the high dose.

(2) Subject population studied and planned sample size

The subject population consisted of patients with non-arteritic central retinal artery occlusion in the acute phase after the onset (having a diagnosis of non-arteritic central retinal artery occlusion that was between 3 and 48 hours after the onset at the time of informed consent and having a study eye with a decimal visual acuity of ≤ 0.1 and hand motion or better).

The planned sample size was a maximum of 9 subjects, 3 subjects in the low dose group and 6 subjects in the high dose group.

(3) Level and method of blinding

This study was planned and conducted in an open-label fashion.

(4) Kind of control(s) and study configuration

No control group was included in this study.

(5) Method of assignment to treatment

No randomization was planned in this study.

(6) Sequence and duration of all study periods

The study flow is shown in Table 7.1-1.

Low dose: 3 subjects

Explanation/informed consent

Pre-entry examination

Registration

Days 1 to 3　　　　Study treatment

	KUS121: 25 µg/eye
Repeated intravitreal administration for 3 days	
Days 4, 6, and 8	Assessment
Week 2	Assessment
Weeks 4 and 8	Assessment
Week 12	Final observation
DSMC	
Discussion on progression to the next cohort	
High dose: 6 subjects	
Explanation/informed consent	
Pre-entry examination	
Registration	
Days 1 to 3	Study treatment
	KUS121: 50 µg/eye
Repeated intravitreal administration for 3 days	
Days 4, 6, and 8	Assessment
Week 2	Assessment
Weeks 4 and 8	Assessment
Week 12	Final observation

Table 7.1-1 Study Flow

(7) Safety, data monitoring or special steering or evaluation committees

The DSMC was founded in this study [see Section 4. (4)].

(8) Interim analyses

No interim analysis was planned in this study.

7.2 Discussion of Study Design, Including the Choice of Control Groups

Since the investigational drug is a new active ingredient with neither the safety nor efficacy confirmed in humans, this study was designed to administer it at the high dose only after carefully confirming the safety at the low dose. Accordingly, the sample size was a maximum of 9 subjects, 3 subjects in the low dose (25 µg) group and 6 subjects in the high dose (50 µg) group. Prior to progression from the low dose group to the high dose group, the DSMC held a meeting to discuss whether to continue the study in consideration of objective opinions of third parties. Since the annual incidence rate of central retinal artery occlusion is as low as 0.7 to 1.8 per 100,000 persons[14)-16)], it seemed very difficult to recruit target patients in this study; therefore, the

target sample size was 9 subjects.

Since the number of patients is limited and intravitreal administration of placebo, instead of the investigational drug, is ethically inappropriate, no control group was included.

7.3 Selection of Study Population

Patients meeting all of the inclusion criteria and none of the exclusion criteria were eligible to participate in the study.

7.3.1 Inclusion Criteria

(1) The patient is 20 years or older at the time of informed consent.
The patient has a diagnosis of non-arteritic central retinal artery occlusion that is between 3 and 48 hours after the onset at the time of informed consent.

(2) The patient has a study eye with a decimal visual acuity of ≤ 0.1 and hand motion or better.

(3) The patient meets all of the following at the pre-entry examination:
Hemoglobin ≥ 10 g/dL
White blood cell count ≥ 2,000 /μL
Platelet count ≥ 100,000/μL
AST ≤ 50 IU/L
ALT ≤ 50 IU/L
Total bilirubin ≤ 1.5 mg/dL
Creatinine ≤ 1.5 mg/dL

(4) The male patient agrees to avoid pregnancy from the start of study treatment to 4 weeks after the last dose of study treatment.
The female patient is postmenopausal (at least 1 year after the last menstrual period) or has undergone surgical sterilization such as bilateral oophorectomy.

(5) The patient has provided written informed consent to participate in the study.

[Rationale]
(2) and (3) These criteria were specified to select patients with non-arteritic central retinal artery occlusion in the acute phase after the onset, the target disease of this study.
(1) and (4) These criteria were specified to ensure the subject safety.
(5) Since the embryo-fetal developmental safety is unknown, this criterion was specified.
(6) This criterion was specified to conduct the study ethically.

7.3.2 Exclusion Criteria

(1) The patient has any of the following diseases concurrently:
Serious heart, liver, or kidney disease
Poorly controlled diabetes mellitus

(2) The patient has a study eye meeting any of the following:

Ocular or periocular infection (including possible infection)

Uncontrollable intraocular pressure despite intraocular pressure-lowering therapy

Retinal vein occlusion

Abnormal macula

Previous photocoagulation in the macula

Optic media opacity precluding funduscopy

(3) The patient has a fellow eye with a decimal visual acuity of ≤ 0.1 or at least hemi-visual field defect as determined by Goldmann perimetry (hereinafter GP) for the I4e isopter.

(4) The patient has central retinal artery occlusion in both eyes on the same day.

(5) The patient is being treated with immunosuppressants or systemic steroids.

(6) The patient has participated in another clinical study within 16 weeks before informed consent (eligible unless treated with an investigational drug).

(7) The patient cannot understand the written information for informed consent due to mental disease, etc., or visit the study site as specified.

(8) The patient, in the opinion of the attending investigator, has hypersensitivity to fluorescent dye or has a history of hypersensitivity to any protocol-specified drug (detergents, disinfectants, anesthetics, and antimicrobials) for which no alternative is available.

(9) The patient, in the opinion of the investigator or subinvestigator, is ineligible for the study for any other reason.

[Rationale]
(1) to (4) These criteria were specified to conduct the study and make assessments safely.
(5), (6), and (8) These criteria were specified to conduct the study safely.
(7) and (9) These criteria were specified to conduct the study safely and ethically.

7.3.3 Removal of Subjects from Therapy or Assessment

7.3.3.1 Criteria for Discontinuation of Study Treatment

If any of the criteria listed below was met, the investigator or subinvestigator promptly informed the relevant subject, discontinued study treatment, and gave appropriate treatment as needed. In the event of an adverse event, the subject's safety was assured, for instance, by performing examinations. In principle, the subject was observed and examined as specified in Section 7.5.1.1 , "Observation/Examination/Report Items and Schedule" after discontinuation of study treatment to confirm the safety. This was not mandatory if the schedule could not be followed due to transfer to another hospital, etc. After discontinuation, post-study treatment could be started once the adverse event was confirmed to have resolved or stabilized.

(1) Study treatment cannot be started within 24 hours after registration.

(2) The investigator or subinvestigator determines that study treatment cannot be administered due to an adverse event.
(3) The subject wishes for discontinuation of study treatment.
(4) Major protocol violation is found.
(5) The subject dies.
(6) The subject is found to be ineligible for the study after registration.
(7) The investigator or subinvestigator determines that continued participation in the study is inappropriate due to any other reason.

[Rationale]
(1) Since a delay in treatment may reduce the efficacy, the interval between registration and start of treatment was specified.
(2) to (4), (6), and (7) These criteria were specified to conduct the study ethically and safely.
(5) This criterion was specified due to the impossibility of continued participation in the study.

7.3.3.2 Post-Study Treatment

Post-study treatment following completion or discontinuation of the study was not restricted.

[Rationale]
After completion or discontinuation of the study, post-study treatment was allowed without restriction to minimize the suffering of subjects.

7.3.3.3 Contraception and Actions to Be Taken in Case of Pregnancy

Male subjects were required to avoid pregnancy from the first day of study treatment to 4 weeks after the last day of study treatment. Reliable contraceptive methods such as barrier contraceptives (condom use for contraception) or oral contraceptives (contraceptive pills for partners of male subjects) had to be selected.

Any subject whose partner became pregnant between the first day of study treatment and 4 weeks after the last day of study treatment was required to promptly contact his attending investigator. The attending investigator reported the pregnancy to the investigator. The attending investigator collected information on the pregnancy, childbirth, fetus, and newborn baby and reported the information to the head of Kyoto University Hospital through the investigator.

There was no report of pregnancy, childbirth, fetus, or newborn baby of any subject’s partner.

[Rationale]
While no histopathological effect of KUS121 on reproductive organs was observed in general toxicity studies, no reproductive toxicity study has been conducted, leaving its effect on reproductive development unknown. After administration of the investigational drug, KUS121 is expected to be only minimally (0.1 to 0.2 ng/mL) distributed to blood and then rapidly eliminated; therefore, any effect of KUS121 on reproductive development may be prevented by avoiding pregnancy until 4 weeks after the last day of study treatment.

7.4 Treatments

7.4.1 Treatments Administered

In this study, subjects with non-arteritic central retinal artery occlusion were intravitreally treated with the investigational drug for the study eye once daily for 3. Study treatment was started within 24 hours after registration.

Two cohorts, low (25 μg) and high doses (50 μg), were included. Progression to the next cohort was specified in Section7.4.1.4 , "Decision to Progress to the Next Cohort."

In each cohort, the second and subsequent subjects were treated after the investigator confirmed the safety based on data collected from the first subject for 2 weeks after repeated administration for 3 days.

7.4.1.1 Premedication

With the first day of treatment as Day 1, the investigational drug was administered from Days 1 to 3. The investigational drug was administered 22 to 26 hours after the previous dose on Days 2 and 3.

Preoperatively, a new quinolone antibiotic (gatifloxacin or moxifloxacin) for ocular instillation was used approximately 4 times daily for antibacterial purposes. For subjects with a history of allergy to new quinolone antibiotics, broad-spectrum antibiotics for ocular instillation such as cephems (0.5% cefmenoxime) were instead used.

Before administration of the investigational drug, the conjunctival sac was washed with 0.26% povidone-iodine solution, and 10% povidone-iodine solution was applied to the eyelid, lid margin, and eyelashes for disinfection. For subjects with hypersensitivity to iodine, an equally effective alternative (chlorhexidine) was instead used.

7.4.1.2 Administration of the Investigational Drug

Under local anesthesia with 4% lidocaine for ocular instillation not containing epinephrine, a 30-gauge needle was inserted into the ciliary ring and then the vitreous body to infuse the investigational drug slowly with the needle tip confirmed to be deep in the vitreous body under an operating microscope. For subjects with hypersensitivity to lidocaine, oxybuprocaine was instead used.

7.4.1.3 Post-Operative Treatment

After intravitreal injection, the insertion site was checked for any leakage of the drug solution, and intraocular pressure was measured in the supine position without postural changes. If intraocular pressure was higher by at least 5 mmHg than the preoperative level, anterior chamber paracentesis was performed to reduce intraocular pressure to the preoperative level. A new quinolone antibiotic ointment (0.3% ofloxacin ophthalmic ointment) was applied.

Postoperatively, a broad-spectrum new quinolone antibiotic (gatifloxacin or moxifloxacin) for ocular instillation was used 4 times daily for 3 days. For subjects with a history of allergy to new quinolone

antibiotics, broad-spectrum antibiotics for ocular instillation such as cephems (0.5% cefmenoxime) were instead used.

The supine position was avoided for 2 hours post-dose unless required for examinations.

7.4.1.4 Decision to Progress to the Next Cohort

After the examinations and observations at Week 2 after the first dose or at discontinuation of study treatment were completed for all subjects in the low dose cohort, and verification of source documents versus case report forms for the collected data through direct inspection was then completed, the DSMC discussed the progression to the next cohort. Patient registration was temporarily suspended until the discussion was completed.

The DSMC confirmed that none of the safety problems listed below had occurred in each cohort and no other problems precluding the progression to the next cohort had occurred; if any problem was detected, the DSMC discussed the progression to the high dose cohort in view of safety.

<Safety problems>

(1) Visual function-related adverse events

Non-disease-associated abnormal retinal findings revealed by imaging test such as funduscopy or optical coherence tomography (OCT) and decrease in decimal logMAR visual acuity of ≥ 0.3

(2) Serious adverse events

Excluding cerebrovascular lesions, cardiovascular lesions, and fall, for which a causal relationship to KUS121 can be ruled out

The procedure for discussion by the DSMC was separately specified in documented procedures for the DSMC.

[Rationale]

In a repeated dose toxicity study in monkeys (3-day intravitreal dose toxicity study in cynomolgus monkeys in Section 6.2.1 in the investigator's brochure, Study No.: SBL704-027), abnormal findings in ocular tissues were observed in the outer retinal layer and the central fovea. Therefore, local ocular adverse events expected to result in serious disorder were specified in (1) above as the criterion for cancelling the progression to the next cohort in the event of abnormal findings together with reduction in visual acuity.

First, the reason for using data collected for 2 weeks after treatment is explained below. After single administration of KUS121 to cynomolgus monkeys at a dose of 100 μg/eye, the KUS121 concentration in the retina/choroid was 28000 ng/g at 2 hours post-dose and 478 ng/g at 26 hours post-dose, which was 50 times lower than at 2 hours post-dose. Hence, since KUS121 is not expected to remain in the eye at potentially toxic concentrations on the day after administration in patients, adverse drug reactions may be detected within 1 week after the start of treatment. In the repeated dose toxicity study in cynomolgus monkeys, in fact, fundal discoloration on Day 3 in the high dose group, which was observed in 1 of 3 animals, resolved on Day 7 or 15, and abnormal OCT findings on Day 3 were resolving on Day 15, indicating that it is very unlikely that

new toxic findings occur beyond 1 week after treatment.
Among abnormal findings in ocular tissues in the repeated dose toxicity study in monkeys (3-day intravitreal dose toxicity study in cynomolgus monkeys in Section 6.2.1 in the investigator's brochure, Study No.: SBL704-027), macular findings were detected as abnormal findings in all eyes examined by funduscopy and also all eyes examined by OCT. In addition, apoptosis findings in the non-macular inner retinal layer were recognized as irregularity of the inner retinal layer/high reflection in the inner nuclear layer on OCT images. Patients with central retinal artery occlusion have edema in the inner retinal layer, followed by apoptosis in the inner retinal layer. However, since the central fovea is rarely affected, and it is practically impossible that edema in the inner retinal layer increases 3 days to 1 week after the onset compared with several hours after the onset (before treatment) or irregularity of the inner retinal layer/high reflection in the inner nuclear layer occurs, any toxic finding related to KUS121 can be accurately and closely identified as abnormal finding by comparing OCT images obtained before and after treatment. In other words, local ocular toxic findings related to KUS121 may be immediately identified by funduscopy and OCT.
Non-ocular systemic adverse events were specified in (2) above as the criterion for cancelling the progression to the next cohort. In the present study, it is difficult to examine the general physical condition of each subject completely, given limited time between onset and registration. On the other hand, high morbidity and mortality rates of cerebrovascular events due to generalized arteriosclerosis have been reported in patients with central retinal artery occlusion[17)]. In addition, since quite elderly patients are expected to be enrolled, there may be many complications and concurrent conditions related to arteriosclerotic disease. Conditions due to underlying disease and clearly unrelated to the study were excluded from the criterion for cancelling the progression to the next cohort.

7.4.2 Identity of Investigational Products

KUS121 is a compound with a molecular weight of 458.44 and a sodium salt of aminonaphthalenesulfonic acid derivative 【chemical name (nomenclature system): Sodium 4-Amino-3-[6-(4-fluoro-2-methylphenyl) pyridine-3-ylazo]naphthalene-1-sulfonate (IUPAC)】. It is a dark red crystalline solid, and its aqueous solution is red.

7.4.2.1 Overview of the Investigational Drug (Drug Product)

Investigational ingredient code: KUS121
Description: light red or light red/white patchy powder or mass
Formulation for 0.025 mg (25 μg): Lyophilized injection containing 0.05 mg of KUS121 per vial
Dissolved in 200 μL of water for injection to use 100 μL of the solution
Serial No.: KUF 1601
Expiration date: 9 March 2018
Formulation for 0.05 mg (50 μg): Lyophilized injection containing 0.1 mg of KUS121 per vial
Dissolved in 200 μL of water for injection to use 100 μL of the solution
Serial No.: KUF 1602
Expiration date: 13 September 2018

Storage conditions: Store in an air-tight container, protected from light, at room temperature.

Packaging: Vials for the investigational drug are labeled, and 10 vials are packaged per partitioned paper box.

Labeling:

<Vial label>

For clinical trial
Lyophilized formulation of KUS121 for intravitreal injection (for 0.025 mg)
ID code: KUS121
Containing 0.05 mg of KUS121 per vial
Serial No.: KUF 1601
Sponsor investigator: Hanako Ikeda
Associate Professor, Institute for Advancement of Clinical and Translational Science, Kyoto University Hospital

For clinical trial
Lyophilized formulation of KUS121 for intravitreal injection (for 0.05 mg)
ID code: KUS121
Containing 0.1 mg of KUS121 per vial
Serial No.: KUF 1602
Sponsor investigator: Hanako Ikeda
Associate Professor, Institute for Advancement of Clinical and Translational Science, Kyoto University Hospital

<Box label>

For clinical trial
Lyophilized formulation of KUS121 for intravitreal injection (for 0.025 mg)
ID code: KUS121
Containing 0.05 mg of KUS121 per vial
Serial No.: KUF 1601
Storage conditions: Room temperature
Expiration date: Specified in documented procedures for handling the investigational drug
Sponsor investigator: Hanako Ikeda
Associate Professor, Institute for Advancement of Clinical and Translational Science, Kyoto University Hospital
54 Shogoin-Kawahara-cho, Sakyo-ku, Kyoto
Quantity: 10 vials

For clinical trial
Lyophilized formulation of KUS121 for intravitreal injection (for 0.05 mg)
ID code: KUS121
Containing 0.1 mg of KUS121 per vial
Serial No.: KUF 1602
Storage conditions: Room temperature
Expiration date: Specified in documented procedures for handling the investigational drug

Sponsor investigator: Hanako Ikeda
Associate Professor, Institute for Advancement of Clinical and Translational Science, Kyoto University Hospital
54 Shogoin-Kawahara-cho, Sakyo-ku, Kyoto
Quantity: 10 vials

7.4.2.2 Management of the Investigational Drug

The investigational drug manager appropriately stored and managed the investigational drug in accordance with the documented procedures for managing the investigational drug, and prepared an investigational drug accountability log.

7.4.3 Method of Assigning Subjects to Treatment Groups

Not applicable because this study was a Phase I/II, open-label, dose-escalation, two-cohort study. The Web registration system was used to register patients.

7.4.3.1 Collection of Information on the Study Site and Prior Assignment of ID Codes/Passwords

The data center confirmed information on the study site, made an arrangement to start Web registration, and informed the investigator that patient registration became acceptable. Unique ID codes and passwords were assigned to the investigator, subinvestigator, and study collaborator when registered as users. The investigator, subinvestigator, and study collaborator appropriately managed the assigned ID codes and passwords, and registered patients.

7.4.3.2 Patient Registration

(1) The investigator or subinvestigator obtained written informed consent from each potential subject and confirmed his/her eligibility for the study.

(2) After confirming through screening that the potential subject met all of the inclusion criteria and none of the exclusion criteria, the investigator or subinvestigator accessed the study-specific Web registration system through the Internet to enter necessary information for patient registration (available 24 hours a day every day, including weekends and holidays).

(3) For the potential subject determined to be eligible for the study on the Web registration system, registration results and the registration number were displayed. The investigator or subinvestigator confirmed the registration results and started protocol-specified study treatment. The registration results were also communicated to the investigator or subinvestigator through e-mail. The investigator, subinvestigator, or study collaborator filled in the registration number in the subject screening log.

(4) For the potential subject determined to be ineligible for the study on the Web registration system,

the investigator or subinvestigator informed him/her that he/she could not be registered in the study. The investigator or subinvestigator filled in the fact of ineligibility and the reason in the electronic medical chart. The ineligible potential subject was not registered on the Web registration system.

(5) If the study collaborator helped in the operation described in (2) to (3), the investigator or subinvestigator filled out a patient registration card in advance to confirm the eligibility, the study collaborator performed the operation described in (2) to (3) in accordance with the patient registration card, and the investigator or subinvestigator confirmed registration results. The patient registration card was appropriately retained after registration.

The investigator or subinvestigator was not allowed to start protocol-specified study treatment until patient registration was completed.

Contact for Web registration:
The URL and ID code were sent to each user after patient registration became acceptable.
Available 24 hours a day (including weekends and holidays)

Contact information for questions about operations for Web registration:
Department of Data Science, Institute for Advancement of Clinical and Translational Science, Kyoto University Hospital
Tel: 075-751-3397/3398 (for inquiries)
E-mail: ds_dm@ml.kuhp.kyoto-u.ac.jp (for inquiries)
Available at 9:00 to 17:00 on Monday through Friday
[excluding weekends, holidays, the anniversary of Kyoto University (18 June), summer holidays, and New Year holidays]

7.4.3.3 Cautions for Registration

Study treatment was never allowed to precede registration.

If information entered in the Web registration system was not complete, registration was not completed until completeness was achieved.

7.4.4 Selection of Doses in the Study

7.4.4.1 Rationale for the Initial Dose Level

In this study, the dose of the investigational drug was 25 μg/eye for the low dose and 50 μg/eye for the high dose.

Since KUS121 is intravitreally administered, the administration procedure itself is invasive. In addition, KUS121 is indicated for ophthalmologic disease with severe visual dysfunction, almost blindness, for which no treatment is available. Hence, the dose levels and regimen with no concern about local or systemic toxicity

and with potential efficacy in improving visual acuity were selected. Since VCP has a homology of 100% among rats, monkeys, and humans, and plays a role in protein degradation in all body cells in all these species, the effect of KUS121 on VCP may not vary greatly among the species. Furthermore, since humans resemble monkeys in the anatomical structure of the eye, especially retina, optic disc, vitreous body, lens, and eye chamber, although the volume differs, species differences may be insignificant in interpreting results.

7.4.4.1.1 Safety

A unilateral intravitreal dose of 25 µg/eye in humans corresponds to a systemic dose of 0.42 µg/kg (an assumed body weight of 60 kg), which is approximately 71000 times the no-observed adverse effect level (NOAEL) of 30 mg/kg in a repeated intravenous dose study in rats and approximately 400 times the systemically non-toxic dose of 500 µg/3 kg = 167 µg/kg (bilaterally administered at a dose of 250 µg/eye with an assumed body weight of 3 kg) in the 3-day intravitreal dose study in monkeys, with sufficient margins.

The local safety was evaluated by regarding the vitreous body as one compartment and therefore dividing the dose by the vitreous volume in accordance with the guideline[18)]. After unilateral intravitreal administration to humans at a dose of 25 µg/eye, the initial vitreous concentration was calculated to be 25 µg/4.5 mL = 5.56 µg/mL (an assumed vitreous volume of 4.5 mL), which was 6 times lower than the NOAEL of 50 µg/1.5 mL = 33.3 µg/mL (an assumed vitreous volume of 1.5 mL) in the 3-day intravitreal dose study in monkeys. In the 3-day intravitreal dose study in monkeys, very mild hypocellularity and apoptosis findings in the macular retina were observed in 1 of 6 eyes in the 100 µg group, but with no significant reduction in electroretinographic visual function. Anterior chamber opacity and miosis presumably due to inflammation were observed in 2 animals, but were resolving over time in 1 animal, finally with no clear histological evidence of anterior chamber inflammation or iris abnormality in either animal. The initial vitreous concentration in humans was 12 times lower than 100 µg/1.5 mL = 66.7 µg/mL, the dose concentration with minimal changes that had no effect on visual function.

KUS121 inhibited the ATPase activity of N-ethylmaleimide-sensitive fusion protein (NSF) (1 µM), weakly inhibited two phosphoenzymes (10 µM), and bound to multiple receptors and transporters (12 µM) (Sections 4.3.1, 4.3.2, and 4.3.3 in the investigator's brochure). In the 3-day intravitreal dose study in monkeys, however, no histological or electroretinographic functional disorder was observed at a dose of 50 µg/eye, indicating that KUS121 had no toxic effect *in vivo* in these conditions. The initial vitreous concentration in humans at the initial dose level (25 µg/eye) was 6 times lower than that in monkeys at a dose of 50 µg/eye, ensuring a safety margin.

7.4.4.1.2 Pharmacology

While the pharmacokinetic-pharmacodynamic (PK/PD) relationship of the investigational drug has not been studied, KUS121 may act pharmacologically by reaching the retina, the target tissue of the drug, or remaining in the adjacent vitreous body in amounts sufficient to inhibit the ATPase activity.

KUS121 concentrations in human ocular tissues following intravitreal administration, which were estimated using data from an ocular tissue distribution study of KUS121 intravitreally administered to monkeys at a

single dose of 100 µg/eye, are shown in Table 7.4-1. The KUS121 concentration in the retinochoroid was estimated to be 5090 nM at 2 hours after intravitreal administration of KUS121 to humans at a dose of 25 µg/eye. The vitreous concentration in humans was estimated to be 12100 and 353 nM immediately and at 2 hours post-dose, respectively. These concentrations were equal to or higher than the 90% inhibitory concentration (IC_{90}) (964 nM) and 50% inhibitory concentration (IC_{50}) (109 nM) of KUS121 for ATPase activity, indicating that KUS121 was expected to be effective in humans.

Table 7.4-1 Estimated KUS121 Concentrations in the Vitreous Body and Retinochoroid Following Intravitreal Administration of KUS121 to Humans

Monkey/human	Dose (µg/eye)	Tissue	Tissue KUS121 concentration (ng/g or mL, nM in parentheses)		
			Immediately post-dose[c]	2 hours post-dose	26 hours post-dose
Single intravitreal administration in monkeys[a]	100	Vitreous body	66700 (145000)	1940 (4230)	73.0 (159)
		Retinochoroid	-	28000 (61100)	478 (1040)
Estimation in humans following single intravitreal administration[b]	50	Vitreous body	11100 (24200)	323 (705)	12.2 (26.6)
		Retinochoroid	-	4670 (10200)	79.7 (174)
	25	Vitreous body	5560 (12100)	162 (353)	6.10 (13.3)
		Retinochoroid	-	2330 (5090)	39.8 (86.7)

[a] Study No. PBC704-019, from Table 5.3.1-1 in the investigator's brochure

[b] Concentrations were calculated using the following formula with an assumed vitreous volume of 1.5 mL for monkeys and 4.5 mL for humans:

Concentration in human ocular tissue = concentration in monkey ocular tissue × (dose for humans/dose for monkeys) × (1.5/4.5)

[c] The dose was divided by the monkey or human vitreous volume (1.5 or 4.5 mL).

- Not calculated

7.4.4.1.3 Initial Dose Level

In the 3-day intravitreal dose study in monkeys, the toxic findings of special concern were macular foveal edema and neuronal hypocellularity/apoptosis in the inner or outer nuclear layer. These events may be due to vitreous traction caused by physical effects of KUS121 on the gel-like vitreous structure after intravitreal injection of the high concentration solution of KUS121, resulting in damage to retinal ganglion cells, foveal detachment or edema, and damage to photoreceptor cells/neural cells in the inner nuclear layer. This assumption is supported by posterior vitreous detachment in some animals. In addition, the limited localization

of those damages indicated that high local drug concentrations in the posture under anesthesia may be responsible. Given the lower dosing formulation concentration for humans (0.25 mg/mL at the initial dose level of 25 μg/eye) than for monkeys (5.0 mg/mL at a dose of 250 μg/eye with serious disorder and 2.0 mg/mL at a dose of 100 μg/eye with minimal changes that had no effect on visual function), quicker drug diffusion in elderly humans due to advanced vitreous liquefaction and degeneration than in young monkeys, and quicker drug diffusion in the vitreous body in humans due to full-body motion and use of the fellow eye after administration than in monkeys, local ocular concentrations of KUS121 in humans at a dose of 25 μg/eye may be much lower than in monkeys at a dose of 100 μg/eye, with a very low risk of disorder. Advanced vitreous liquefaction and degeneration in elderly humans than in young monkeys also indicate that vitreous traction is less likely in humans. Taken together, vitreous traction or neuronal hypocellularity due to changes in the gel-like vitreous structure may be unlikely in humans.

Central retinal artery occlusion involves death of retinal ganglion cells and/or neural cells in the inner nuclear layer due to ischemia or reperfusion within a few days after the onset. KUS121 may prevent death of retinal ganglion cells and neural cells in the inner nuclear layer, which occurs while it is expected to act pharmacologically, and thereby improve visual acuity and visual field, although slightly.

Central retinal artery occlusion ends in severe and perpetual vision/visual field loss immediately after the onset, with no effective treatment established. In addition, it is difficult to restore retinal function once cells die. Ethically, on the other hand, it must be expected that visual function would improve after invasive intravitreal administration. In these circumstances, the selected initial dose level, which is 6 times lower than the NOAEL, but is unlikely to cause serious toxicity in humans and is expected to improve visual function, may be appropriate.

7.4.4.2 Rationale for Repeated administration Once Daily for 3 Days

After ischemia due to central artery occlusion persists beyond 2 to 6 hours of onset of central retinal artery occlusion, retinal ganglion cells and/or neural cells in the inner nuclear layer begin to die, and subsequent loss of nerve fibers results in rapid inner retinal layer thinning, ending in perpetual visual dysfunction, even if blood flow is restored later[19)]. In fact, blood flow is often restored spontaneously within 1 day, at the latest, within a few days, but reperfusion after a certain time of ischemia is reportedly harmful to neural cells, resulting in further cell death even after revascularization. Clinical symptoms are vision/visual field loss due to retinal cell dysfunction as well as ischemia, and cell death, which occurs in the following few days, perpetuates the vision/visual field loss.

In an experiment of central retinal artery occlusion induced in monkeys, many vacuoles and large mitochondria in retinal ganglion cells at 3.5 hours after the start of ischemia, and loss of cytoplasm and pyknosis in most ganglion cells at 16 hours were reported[20)]. In an embolism experiment in pigs, advanced degeneration of ganglion cells after 5 hours of ischemia and almost complete degeneration after 3 days were reported[21)]. In rats with ischemia-reperfusion impairment, a model of central retinal artery occlusion, it was reported that the number of retinal ganglion cells decreased rapidly from 6 to 24 hours after ischemia and then slightly until 72 hours after ischemia, but hardly decreased thereafter until 5 days after ischemia[22)]. Given

various reports, including those mentioned above, it was assumed that ischemia-induced cell death would peak at 6 to 24 hours after ischemia and hardly occur after 3 to 5 days in humans as well.

Also given little change in visual acuity after 1 week of onset in many clinical course reports, the findings from the aforementioned animal experiments may be applicable to humans. Since blood flow is usually restored spontaneously within 1 day after central retinal artery occlusion, at the latest, within a few days, treatment with KUS121 for 3 to 4 days after the onset may be appropriate as cytoprotective treatment intended to maintain reversible cellular function for a longer period of time. Accordingly, it was assumed that maximum effect would be achieved after repeated administration for 3 days.

To measure KUS121 concentrations in plasma and ocular tissues (retina/choroid, aqueous humor, and vitreous body) after single intravitreal administration of KUS121 to a cynomolgus monkey, on the other hand, the animal intravitreally received the first dose of KUS121 in the left eye at a dose of 100 μg/eye and the second dose in the right eye 24 hours later. Both eyes were removed 2 hours after the second dose (26 hours after the first dose), and KUS121 concentrations in ocular tissues were measured using liquid chromatograph-tandem mass spectrometer (LC/MS/MS). The vitreous KUS121 concentration was 1940 ng/mL at 2 hours post-dose and rapidly decreased to 73 ng/mL 26 hours post-dose.

Furthermore, since KUS121 is intravitreally administered, the safety of the injection procedure must be ensured. In general, the previous administration site is avoided when intravitreal injection is given to patients within 1 month after the previous injection. Hence, continuous administration of up to 3 doses may be acceptable to prevent adverse reactions to the injection procedure itself. In addition, a twice-daily regimen or a more intensive regimen is not practical with a higher risk of infection.

In conclusion, intravitreal administration once daily for 3 consecutive days may be necessary and sufficient to achieve maximum effect without procedure problems in patients.

7.4.5 Selection and Timing of Dose for Each Subject

In this study, progression to the high dose cohort was made only after confirming the safety in the low dose cohort to ensure the subject safety. Progression to the next cohort is detained in Section 7.4.1.4 , "Decision to Progress to the Next Cohort."

7.4.6 Blinding

This study was planned and conducted in an open-label fashion.

7.4.7 Prior and Concomitant Therapies

7.4.7.1 Prohibited Medications and Therapies

The concomitant use of the following was prohibited during the treatment period:

- Drug therapies for non-arteritic central retinal artery occlusion not listed in Section 7.4.7.3 , "Acceptable Concomitant Medications and Recommended Supportive Cares"

- Eye massage
- Hyperbaric oxygen therapy
- Carbon dioxide inhalation
- All other investigational drugs and products

[Rationale]
These were specified, because they may affect the safety or efficacy evaluation of the investigational drug.

7.4.7.2 Medications Requiring Careful Use

In this study, no medication or therapy requiring careful use was specified.

7.4.7.3 Acceptable Concomitant Medications and Recommended Supportive Cares

(1) Ocular instillation of intraocular pressure-lowering agents: During the screening, treatment, and observation periods, intraocular pressure-lowering agents [prostaglandin preparations (latanoprost)] were used if intraocular pressure was 22 mmHg or higher.

(2) Administration of carbonate dehydratase inhibitors: Intraocular pressure was measured 30 (±15) minutes after intravitreal injection. If intraocular pressure was 30 mmHg or higher, intraocular pressure was measured 30 minutes (±15 minutes) later again. If intraocular pressure still remained 30 mmHg or higher, intravenous infusion of carbonate dehydratase inhibitor 500 mg was considered.

(3) Anterior chamber paracentesis: Intraocular pressure was measured immediately after intravitreal injection. If intraocular pressure was higher by at least 5 mmHg than the preoperative level, anterior chamber paracentesis was performed, and recovery of intraocular pressure to the preoperative level was confirmed. If intraocular pressure still remained 40 mmHg or higher despite the treatment described in (2) after intravitreal injection, anterior chamber paracentesis was performed in fear of irreversible damage to visual function caused by increased intraocular pressure.
After the treatments described in (2) and (3), intraocular pressure was frequently measured and followed up until it was reduced to 30 mmHg or lower.

(4) For intense inflammation following study treatment (anterior chamber cell: Grade 2 or higher, anterior chamber flare: Grade 2 or higher), steroid for ocular instillation (betamethasone) was used 3 to 6 times a day.

Any drug or therapy used for concurrent disease before the start of the study was not restricted during the treatment period, unless specified as prohibited medication or therapy.

[Rationale]
These were specified to assure the safety and ethicality of the study for subjects. The treatments for increased intraocular pressure described in (1) to (3) were specified in fear of irreversible damage to the inner retinal

layer, which is especially vulnerable in this disease, caused by increased intraocular pressure, even of short duration. The treatment for inflammation described in (4) was also specified in fear of irreversible damage to the inner retinal layer, which is more vulnerable in this disease than usual, caused by prolonged inflammation.

7.4.8 Treatment Compliance

Since KUS121 is intravitreally administered, the administration procedure itself is invasive. Therefore, details of premedication, administration method, and post-operative treatment for administration of KUS121 were separately specified in documented procedures.

In this study, the items listed below were documented, and the monitor confirmed the compliance of treatment status through direct inspection.

- Study treatment status in the case report form completed by the investigator or subinvestigator (see Appendix 16.1.2)
- Investigational drug accountability log prepared by the investigational drug manager

7.5 Efficacy and Safety Variables

7.5.1 Efficacy and Safety Measurements Assessed and Flow Chart

The study schedule is shown in Table 7.5-1.

Table 7.5-1 Study Schedule

Timing	Before registration	Study treatment period																				
Day	Day 0 or 1	Day 1							Day 2							Day 3						
Time		Pre-dose	Dosing	Post-dose					Pre-dose	Dosing	Post-dose					Pre-dose	Dosing	Post-dose				
				30 min	1 h	2 h	4 h	6 h			30 min	1 h	2 h	4 h	6 h			30 min	1 h	2 h	4 h	6 h
Study treatment			●							●							●					
Written informed consent	○																					
Information on baseline subject characteristics/target disease	○																					
Information on concomitant therapy	○		◄																			►
Information on study treatment			○							○							○					
Physical findings (height/weight)	○																					
Physical findings (blood pressure/pulse rate)	○			○		○	○	○			○		○	○	○			○		○	○	○
Physical findings (body temperature)	○				○			○				○			○				○			○
Subjective/objective symptoms	○		◄																			►
Laboratory tests	○												○							○		
Urinalysis	○												○3							○3		
Plasma drug concentration						○	○		○				○	○		○				○	○	
ECG	○					○							△2							△2		
Chest x-ray	○																					
Decimal visual acuity test	○								○							○						

ETDRS visual acuity test	○								○							○						
GP	○																					
Slit-lamp examination	○			○					○		○					○		○				
Intraocular pressure	○		○1	○					○	○1	○					○	○1	○				
Retinal sensitivity	○																					
ERG	○																					
OCT	○								○							○						
Funduscopy/fundus photography	○								○							○						
Fluorescein angiography	○																					
Laser speckle flowgraphy	○								○							○						

1: Performed immediately after administration of the investigational drug 2: When abnormality is detected after surgery on Day 1 3: For the first urine after administration, in principle, with no sampling time specified

Timing	Observation period							Discontinuation
Day	Day 4	Day 6 (5-7)	Day 8 (7-9)	Week 2 (14-18)	Week 4 (28-32)	Week 8 ± 1 week	Week 12 ± 2 weeks	At discontinuation
Information on concomitant therapy	←	—	—	—	—	→		
Physical findings (blood pressure/pulse rate)	○		○	○				○
Physical findings (body temperature)	○							
Subjective/objective symptoms	←	—	—	—	—	→		○
Laboratory tests	○2			○				○
Urinalysis	○1							
Plasma drug concentration	○2							
Decimal visual acuity test	○		○	○	○	○	○	○
ETDRS visual acuity test	○		○	○	○	○	○	○
GP	○		○	○	○		○	○
Slit-lamp examination	○		○	○	○	○	○	○
Intraocular pressure	○		○	○	○	○	○	○
Retinal sensitivity	○		○	○	○		○	○
ERG				○	○		○	○
OCT	○		○	○	○	○	○	○
Funduscopy/fundus photography	○		○	○	○	○	○	○
Fluorescein angiography		○						
Laser speckle flowgraphy		○			○		○	

1: Urine sampling at 24 hours (±2 hours) post-dose, in principle 2: 24 hours (±2 hours) post-dose

7.5.1.1 Observation/Examination/Report Items and Schedule

7.5.1.1.1 Study Period

The study period for individual subjects was from registration to the day of the final observation at Week 12 after treatment or 14 days after discontinuation.

Of this period, the study treatment period was from registration to Day 3 of treatment, and the observation period was from the day after Day 3 of treatment from Week 12.

7.5.1.1.2 Examination/Observation Items from before Registration to Day 1

7.5.1.1.2.1 Examination/Observation Items from before Registration to before Administration on Day 1

After written informed consent was obtained from each patient, the items listed below were examined or observed.

For chest x-ray examination and 12-lead rest electrocardiography (ECG), results obtained before informed consent could be used to reduce subject's burden if they were obtained within 28 days before registration. For HbA1c (NGSP), results obtained before informed consent could be used if they were obtained within 1 week before registration. For all other items, results obtained after the onset of the target disease could be used. The Early Treatment Diabetic Retinopathy Study (ETDRS) visual acuity test, OCT, retinal sensitivity, electroretinography (ERG), fluorescein angiography, and laser speckle flowgraphy were performed no later than administration, and all other examinations were performed before registration.

(1) Baseline subject characteristics: birth date, sex, date of informed consent, previous disease, concurrent disease, allergy, and smoking history
(2) Information on the target disease: date of initial diagnosis, diagnosis, affected eye, time of onset, presence or absence of cilioretinal artery, and information on prior treatment
(3) Information on concomitant therapy: name of drug/treatment
(4) Physical findings: height, weight, blood pressure, pulse rate, and body temperature
(5) 12-lead rest ECG: presence or absence of abnormality and findings
(6) Chest x-ray: presence or absence of abnormality
(7) Laboratory tests

Hematology: red blood cell count, hemoglobin, hematocrit, white blood cell count, platelet count, and differential leukocyte count (neutrophil, lymphocyte, eosinophil, basophil, and monocyte)

Blood chemistry: AST, ALT, LDH, alkaline phosphatase, total protein, albumin, total bilirubin, creatinine, BUN, uric acid, CK, blood glucose, HbA1c*, Na, K, Cl, and Ca

Immunology: C-reactive protein (CRP)

* Not examined at emergency testing

(8) Urinalysis: urinary occult blood, urinary protein, and ketone

(9) Visual acuity test: decimal visual acuity (both eyes) and ETDRS visual acuity (study eye)
(10) Visual field test (both eyes): GP
(11) Slit-lamp examination (study eye): presence or absence of abnormality
(12) Intraocular pressure (study eye)
(13) OCT (both eyes): total retinal thickness, presence or absence of abnormality, and findings
(14) Retinal sensitivity (study eye)
(15) Funduscopy (study eye): presence or absence of abnormality, findings, and fundus photography
(16) Fluorescein angiography (study eye)
(17) Laser speckle flowgraphy (study eye): blood flow rate
(18) ERG (both eyes)

7.5.1.1.2.2 Examination/Observation Items after Administration on Day 1

(1) Information on study treatment: day and time of administration, dose, treated eye, and practitioner (initials)
(2) Information on concomitant therapy: name of drug/treatment and duration of treatment
(3) Physical findings: blood pressure, pulse rate [30 minutes (±10 minutes), 2 hours (±15 minutes), 4 hours (±15 minutes), and 6 hours (±15 minutes) post-dose], and body temperature [1 hour (±15 minutes) and 6 hours (±15 minutes) post-dose]
(4) Plasma drug concentration [2 hours (±15 minutes) and 4 hours (±15 minutes) post-dose]
(5) 12-lead rest ECG [2 hours (±15 minutes) post-dose]
(6) Subjective/objective symptoms: presence or absence of adverse event/adverse drug reaction, event term, date of onset, severity, seriousness, causal relationship, treatment, outcome, and date of outcome assessment
(7) Slit-lamp examination [study eye, 30 minutes (±15 minutes) post-dose]: presence or absence of abnormality
(8) Intraocular pressure [study eye, immediately and 30 minutes (±15 minutes) post-dose]

7.5.1.1.3 Examination/Observation Items on Days 2 and 3

(Ophthalmologic examination is specified only for the study eye)

7.5.1.1.3.1 Examination/Observation Items before Administration

(1) Plasma drug concentration [before administration = 24 hours (±2 hours) after the previous dose]
(2) Visual acuity test: decimal visual acuity and ETDRS visual acuity
(3) Slit-lamp examination: presence or absence of abnormality
(4) Intraocular pressure
(5) OCT: presence or absence of abnormality and findings

(6) Funduscopy: presence or absence of abnormality, findings, and fundus photography

(7) Laser speckle flowgraphy: blood flow rate

(8) Subjective/objective symptoms: presence or absence of adverse event/adverse drug reaction, event term, date of onset, severity, seriousness, causal relationship, treatment, outcome, and date of outcome assessment

7.5.1.1.3.2 Examination/Observation Items after Administration

(1) Information on study treatment: day and time of administration, dose, treated eye, practitioner (initials), and presence or absence of completed administration of the investigational drug

(2) Information on concomitant therapy: name of drug/treatment and duration of treatment

(3) Physical findings: blood pressure, pulse rate [immediately pre-dose and 30 minutes (±10 minutes), 2 hours (±15 minutes), 4 hours (±15 minutes), and 6 hours (±15 minutes) post-dose], and body temperature [1 hour (±15 minutes) and 6 hours (±15 minutes) post-dose]

(4) Plasma drug concentration [2 hours (±15 minutes) and 4 hours (±15 minutes) post-dose]

(5) 12-lead rest ECG [2 hours (±15 minutes) post-dose when abnormality is detected after surgery on Day 1]

(6) Subjective/objective symptoms: presence or absence of adverse event/adverse drug reaction, event term, date of onset, severity, seriousness, causal relationship, treatment, outcome, and date of outcome assessment

(7) Slit-lamp examination [30 minutes (±15 minutes) post-dose]: presence or absence of abnormality

(8) Intraocular pressure [immediately and 30 minutes (±15 minutes) post-dose]

(9) Laboratory tests [2 hours (±15 minutes) post-dose]

Hematology: red blood cell count, hemoglobin, hematocrit, white blood cell count, platelet count, and differential leukocyte count (neutrophil, lymphocyte, eosinophil, basophil, and monocyte)

Blood chemistry: AST, ALT, LDH, alkaline phosphatase, total protein, albumin, total bilirubin, creatinine, BUN, uric acid, CK, blood glucose, Na, K, Cl, and Ca

Immunology: CRP

(10) Urinalysis: urinary occult blood and urinary protein (for the first urine after administration, in principle)

7.5.1.1.4 Examination/Observation Items on Day 4

(Ophthalmologic examination is specified only for the study eye)

(1) Visual acuity test: decimal visual acuity and ETDRS visual acuity

(2) Slit-lamp examination: presence or absence of abnormality

(3) Intraocular pressure

(4) OCT: presence or absence of abnormality and findings

(5) Funduscopy: presence or absence of abnormality, findings, and fundus photography

(6) GP

(7) Retinal sensitivity
(8) Subjective/objective symptoms: presence or absence of adverse event/adverse drug reaction, event term, date of onset, severity, seriousness, causal relationship, treatment, outcome, and date of outcome assessment
(9) Information on concomitant therapy: name of drug/treatment and duration of treatment
(10) Physical findings: blood pressure, pulse rate, and body temperature
(11) Plasma drug concentration [24 hours (±2 hours) post-dose]
(12) Laboratory tests [24 hours (±2 hours) post-dose]
Hematology: red blood cell count, hemoglobin, hematocrit, white blood cell count, platelet count, and differential leukocyte count (neutrophil, lymphocyte, eosinophil, basophil, and monocyte)
Blood chemistry: AST, ALT, LDH, alkaline phosphatase, total protein, albumin, total bilirubin, creatinine, BUN, uric acid, CK, blood glucose, Na, K, Cl, and Ca
Immunology: CRP
(13) Urinalysis: urinary occult blood and urinary protein [urine sampling at 24 hours (±2 hours) post-dose, in principle]

7.5.1.1.5 Examination/Observation Items on Day 6

(Permissible range: Days 5 to 7, ophthalmologic examination is specified only for the study eye)
(1) Subjective/objective symptoms: presence or absence of adverse event/adverse drug reaction, event term, date of onset, severity, seriousness, causal relationship, treatment, outcome, and date of outcome assessment
(2) Information on concomitant therapy: name of drug/treatment and duration of treatment
(3) Fluorescein angiography
(4) Laser speckle flowgraphy: blood flow rate

7.5.1.1.6 Examination/Observation Items on Day 8

(Permissible range: Day7 to 9, ophthalmologic examination is specified only for the study eye)
(1) Visual acuity test: decimal visual acuity and ETDRS visual acuity
(2) Slit-lamp examination: presence or absence of abnormality
(3) Intraocular pressure
(4) OCT: presence or absence of abnormality and findings
(5) Funduscopy: presence or absence of abnormality, findings, and fundus photography
(6) GP
(7) Retinal sensitivity
(8) Subjective/objective symptoms: presence or absence of adverse event/adverse drug reaction, event term, date of onset, severity, seriousness, causal relationship, treatment, outcome, and date of outcome assessment
(9) Information on concomitant therapy: name of drug/treatment and duration of treatment

(10) Physical findings: blood pressure and pulse rate

7.5.1.1.7 Examination/Observation Items at Week 2 (Day 15) after the First Dose

(Permissible range: Days 14 to 18, ophthalmologic examination is specified only for the study eye)

(1) Visual acuity test: decimal visual acuity and ETDRS visual acuity
(2) Slit-lamp examination: presence or absence of abnormality
(3) Intraocular pressure
(4) OCT: presence or absence of abnormality and findings
(5) Funduscopy: presence or absence of abnormality, findings, and fundus photography
(6) GP
(7) Retinal sensitivity
(8) ERG
(9) Subjective/objective symptoms: presence or absence of adverse event/adverse drug reaction, event term, date of onset, severity, seriousness, causal relationship, treatment, outcome, and date of outcome assessment
(10) Information on concomitant therapy: name of drug/treatment and duration of treatment
(11) Physical findings: blood pressure and pulse rate
(12) Laboratory tests

Hematology: red blood cell count, hemoglobin, hematocrit, white blood cell count, platelet count, and differential leukocyte count (neutrophil, lymphocyte, eosinophil, basophil, and monocyte)

Blood chemistry: AST, ALT, LDH, alkaline phosphatase, total protein, albumin, total bilirubin, creatinine, BUN, uric acid, CK, blood glucose, Na, K, Cl, and Ca

Immunology: CRP

7.5.1.1.8 Examination/Observation Items at Week 4 (Day 29)

(Permissible range: Days 28 to 32, ophthalmologic examination is specified only for the study eye)

(1) Visual acuity test: decimal visual acuity and ETDRS visual acuity
(2) Slit-lamp examination: presence or absence of abnormality
(3) Intraocular pressure
(4) OCT: presence or absence of abnormality and findings
(5) Funduscopy: presence or absence of abnormality, findings, and fundus photography
(6) GP
(7) Retinal sensitivity
(8) ERG
(9) Subjective/objective symptoms: presence or absence of adverse event/adverse drug reaction, event term, date of onset, severity, seriousness, causal relationship, treatment, outcome, and date of outcome assessment
(10) Information on concomitant therapy: name of drug/treatment and duration of treatment

(11)　Laser speckle flowgraphy: blood flow rate

7.5.1.1.9 Examination/Observation Items at Week 8

(Permissible range: ±1 week, ophthalmologic examination is specified only for the study eye)

(1)　Visual acuity test: decimal visual acuity and ETDRS visual acuity
(2)　Slit-lamp examination: presence or absence of abnormality
(3)　Intraocular pressure
(4)　OCT: total retinal thickness, presence or absence of abnormality, and findings
(5)　Funduscopy: presence or absence of abnormality, findings, and fundus photography
(6)　Subjective/objective symptoms: presence or absence of adverse event/adverse drug reaction, event term, date of onset, severity, seriousness, causal relationship, treatment, outcome, and date of outcome assessment
(7)　Information on concomitant therapy: name of drug/treatment and duration of treatment

7.5.1.1.10 Examination/Observation Items at Week 12

(Permissible range: ±2 weeks, ophthalmologic examination is specified only for the study eye)

(1)　Visual acuity test: decimal visual acuity and ETDRS visual acuity
(2)　Slit-lamp examination: presence or absence of abnormality
(3)　Intraocular pressure
(4)　OCT: total retinal thickness, presence or absence of abnormality, and findings
(5)　Funduscopy: presence or absence of abnormality, findings, and fundus photography
(6)　GP
(7)　Retinal sensitivity
(8)　ERG
(9)　Subjective/objective symptoms: presence or absence of adverse event/adverse drug reaction, event term, date of onset, severity, seriousness, causal relationship, treatment, outcome, and date of outcome assessment
(10)　Information on concomitant therapy: name of drug/treatment and duration of treatment
(11)　Laser speckle flowgraphy: blood flow rate

7.5.1.1.11 Examination/Observation Items at Discontinuation

(Ophthalmologic examination is specified only for the study eye)

(1)　Visual acuity test: decimal visual acuity and ETDRS visual acuity
(2)　Slit-lamp examination: presence or absence of abnormality
(3)　Intraocular pressure
(4)　OCT: total retinal thickness, presence or absence of abnormality, and findings
(5)　Funduscopy: presence or absence of abnormality, findings, and fundus photography

(6) GP
(7) Retinal sensitivity
(8) ERG
(9) Subjective/objective symptoms: presence or absence of adverse event/adverse drug reaction, event term, date of onset, severity, seriousness, causal relationship, treatment, outcome, and date of outcome assessment
(10) Physical findings: blood pressure and pulse rate
(11) Laboratory tests

Hematology: red blood cell count, hemoglobin, hematocrit, white blood cell count, platelet count, and differential leukocyte count (neutrophil, lymphocyte, eosinophil, basophil, and monocyte)

Blood chemistry: AST, ALT, LDH, alkaline phosphatase, total protein, albumin, total bilirubin, creatinine, BUN, uric acid, CK, blood glucose, Na, K, Cl, and Ca

Immunology: CRP

7.5.1.1.12 Details of Ophthalmologic Examination Items

7.5.1.1.12.1 Decimal Visual Acuity

The visual acuity was measured at a distance of 5 m using a decimal visual acuity chart. For subjects who could not see the largest Landolt ring with a visual acuity of < 0.1, the visual acuity was measured at a gradually decreased distance. For subjects with a visual acuity of < 0.01, the visual acuity was measured as counting fingers, hand motion, light perception, or no light perception. For any probable measurement, a one-level lower value was used as the decimal visual acuity for the study.

7.5.1.1.12.2 ETDRS Visual Acuity

The visual acuity was measured at a distance of 4 m using an ETDRS chart. For subjects with a visual acuity of < 20/160, the visual acuity was measured at a distance of 1 m. The number of letters read and the logMAR visual acuity were determined. The measurement chart was retained with the medical record, etc.

7.5.1.1.12.3 GP

The visual field area was measured by GP for the V4e, I4e, and I2e isopters. The visual field chart was retained with the medical record, etc.

7.5.1.1.12.4 Retinal Sensitivity Test

The retinal sensitivity was measured using Micro Perimeter-3 (MP-3) in the CRAO mode.

7.5.1.1.12.5 OCT

SPECTRALIS was used for macular crossing and raster scanning. The follow-up mode was used.

7.5.1.1.12.6 Fluorescein Angiography

OPTOS or F-10 was used to take pictures of the affected eye. The arm-to-choroid circulation time, arm-to-retina circulation time, and time of maximal venous filling were measured.

7.5.1.1.12.7 Laser Speckle Flowgraphy

The intraocular pressure was simultaneously measured. The mean blur rate (MBR) was measured.

7.5.1.1.12.8 Electroretinography

The flicker latency and amplitude were measured in the mydriatic state using RETeval.

7.5.1.1.13 Plasma Drug Concentration

Blood was collected as specified below to measure plasma drug concentrations.

7.5.1.1.13.1 Blood Collection Tube and Treatment of Blood Specimens

Blood was collected and immediately inverted gently 5 to 10 times. The blood was centrifuged to separate plasma.

The plasma was divided into two microtubes. The microtubes were checked for any loose cap, breakage, or leakage before storing the plasma.

- Blood collection tube: heparin-treated tube
- Volume of collected blood: 2 mL
- Centrifugation: 4°C, 2500 to 3000 rpm for 10 to 15 minutes

7.5.1.1.13.2 Storage and Transport of Specimens

After centrifugation, the plasm was promptly cryopreserved.

7.5.1.1.14 Collection and Storage of Images

Images from the examinations listed below, among those specified in Section 7.5.1.1.2 , “Examination/Observation Items from before Registration to Day 1” to Section 7.5.1.1.11 , “Examination/Observation Items at Discontinuation,” were collected, anonymized, and stored as study data.

(1) GP [from before registration to Day 1, Day 4, Day 8, and Weeks 2, 4, and 12 after the first dose or discontinuation (if applicable)]

(2) OCT, fluorescein angiography, or laser speckle flowgraphy images relating to adverse events (at the onset of the adverse event, in the worst state, or at the final outcome) or causes of adverse events (before or after the onset of the adverse event, etc.)

7.5.1.2 Assessment and Reporting of Adverse Events

7.5.1.2.1 Definitions of Adverse Event and Adverse Drug Reaction

7.5.1.2.1.1 Adverse Events and Adverse Drug Reactions

An adverse event was defined as any untoward medical occurrence in a subject administered an investigational drug. Underlying disease was not handled as an adverse event unless the severity or incidence increased during the study. An adverse drug reaction was defined as any adverse event for which a causal relationship to the investigational drug could not be ruled out.

7.5.1.2.1.2 Seriousness of Adverse Events and Serious Adverse Events

The seriousness of adverse events was categorized as described below.

(1) Not serious
(2) Serious

A serious adverse event was defined as any adverse event specified in Section 7.5.1.2.1.1 , "Adverse Events and Adverse Drug Reactions," that:

(1) Results in death
(2) Is life threatening[*1]
(3) Requires inpatient hospitalization or prolongation of existing hospitalization for the treatment[*2]
(4) Results in persistent or significant disability/incapacity
(5) Leads to a congenital anomaly/birth defect
(6) Is an important medical condition as not specified above[*3]

[*1] This refers to an event in which the subject was at risk of death at the time of the event; it does not refer to an event that hypothetically might have caused death if it were more severe.

[*2] This refers to an adverse event resulting in inpatient hospitalization or prolongation of existing hospitalization to treat the event. It includes hospitalization for the treatment of the adverse event not involving any interventions (rest treatment). It does not include inpatient hospitalization or prolongation of existing hospitalization only for examination purposes, pre-planned hospitalization such as hospitalization for post-study treatment, or follow-up hospitalization for cured or resolving adverse events.

[*3] This refers to an important medical event that may expose the subject to danger or requires intervention to prevent any condition that results in death, is life threatening, requires inpatient hospitalization or prolongation of existing hospitalization for the treatment, or results in persistent or significant disability/incapacity, even though the event is not immediately life threatening or fatal or does not result in hospitalization.

7.5.1.2.1.3 Severity of Adverse Events

The severity of adverse events was categorized as described below.

(1) Mild: no or mild symptoms and not interfering with activities of daily living

(2) Moderate: not severe disorder, but interfering with activities of daily living

(3) Severe: severe disorder and precluding activities of daily living

7.5.1.2.1.4 Assessment of Causal Relationship

The causal relationship of adverse events to the investigational drug or the administration procedure was assessed separately and categorized as described below.

(1) Causal relationship to the investigational drug

Can be ruled out: not reasonably related to the investigational drug

Cannot be ruled out: reasonably related to the investigational drug

(2) Causal relationship to the administration procedure

Can be ruled out: not reasonably related to the administration procedure

Cannot be ruled out: reasonably related to the administration procedure

The causal relationship to the investigational drug or the administration procedure was assessed in consideration of the subject's general condition, concurrent disease, concomitant medication/therapy, and temporal relationship. The causal relationship was assessed by the investigator or subinvestigator.

7.5.1.2.1.5 Outcome of Adverse Events

The outcome of adverse events was categorized as described below.

(1) Recovered: The adverse event has resolved, and the subject has recovered to the original state.

(2) Recovering: The adverse event has almost resolved, and the subject has recovered close to the original state.

(3) Not recovered: The adverse event has not resolved, and the subject is in the same state as at the onset of the adverse event (unchanged).

(4) Recovered with sequelae: The adverse event has resolved, but the subject has sequelae.

(5) Fatal: The subject died (related or unrelated to study treatment)

(6) Unknown: The outcome is unknown with no information available.

7.5.1.2.2 Actions to be Taken for Adverse Events

If an adverse event occurred, the investigator or subinvestigator appropriately treated the relevant subject, reported the event through the case report form, and followed up the event until the end of the study period and further until resolution whenever possible, whether or not it was related to study treatment. All adverse events occurring during the period from the start of study treatment to 14 days after study treatment were followed up, whether or not it was related to study treatment. Any adverse event occurring or worsening after the start of post-study treatment was not assessed in the study. Any adverse event occurring beyond 14 days after study

treatment was followed up whenever possible, if a causal relationship to study treatment (investigational drug or administration procedure) could not be ruled out. This was not mandatory if any symptom resulting from worsening of primary disease or associated with concurrent disease had become chronic or continuous follow-up was precluded due to transfer to another hospital, initiation of post-study treatment, etc.

The investigator or subinvestigator filled out the medical record with information on the adverse event, including findings/symptoms, date of onset, severity, seriousness, causal relationship, presence or absence of treatment (contents if any), and outcome (timing of recovery or symptom stabilization if applicable).

7.5.1.2.3 Expected Adverse Events

Expected adverse reactions to the investigational drug were specified as described below.
(See the investigator's brochure for KUS121 as well.)

7.5.1.2.3.1 Adverse Reactions to the Investigational Drug

In the 3-day repeated intravitreal dose study in monkeys, macular foveal edema, neuronal hypocellularity/apoptosis in the inner or outer nuclear layer, and anterior chamber opacity were observed at doses of 100 μg/eye and higher, and vitreous opacity and posterior vitreous detachment were observed in the 250 μg/eye group.

These results indicated that iritis/endophthalmitis and posterior vitreous detachment/macular hole might occur as adverse reactions to the investigational drug in humans treated with KUS121.

7.5.1.2.3.2 Adverse Reactions Associated with Intravitreal Injection

Ocular hypertension/ocular hypotony, posterior vitreous detachment/retinal tear/retinal detachment/retinal perforation, vitreous hemorrhage, iritis/endophthalmitis, and cataract/lenticular injury could occur in association with intravitreal injection.

7.5.1.2.3.3 Treatment at the Onset of Adverse Drug Reactions

(1) Iritis/endophthalmitis
Anti-inflammatory treatment with steroid was given (details in Section 7.4.7.3 , "Acceptable Concomitant Medications and Recommended Supportive Cares").

(2) Posterior vitreous detachment
Posterior vitreous detachment generally has no effect on visual function, requiring no special treatment.

(3) Macular hole
Macular hole, which may begin to resolve spontaneously approximately 2 weeks after the onset, was only followed up for this period, and treatment with vitreous surgery was considered if it did not begin to resolve thereafter.

(4) Ocular hypertension

Intraocular pressure was reduced with ocular instillation, oral treatment, intravenous infusion, or anterior chamber paracentesis according to intraocular pressure (details in Section 7.4.7.3 , "Acceptable Concomitant Medications and Recommended Supportive Cares").

(5) Retinal tear

Retinal photocoagulation was applied to the site of tear.

(6) Retinal detachment/vitreous hemorrhage

Vitreous surgery was considered depending on the condition.

(7) Cataract, lenticular injury

Cataract surgery was considered if visual function was affected.

If a serious adverse drug reaction occurred and the drug appeared to remain in the vitreous body at high concentrations, vitreous surgery was performed to wash away the drug.

7.5.1.2.3.4 Handling of Deterioration of Visual Acuity

In this study, deterioration of visual acuity as defined below was handled as a serious adverse event.

Vision loss: decrease in decimal logMAR visual acuity > 1.0

In individual subjects, the causal relationship to the investigational drug was assessed based on whether non-disease-associated abnormal ocular findings were revealed by imaging test such as funduscopy or OCT.

7.5.1.2.4 Expedited Reporting of Adverse Events and Related Actions

7.5.1.2.4.1 Report to the Hospital Head, etc. (First Report)

If a serious adverse event (related or unrelated to the investigational drug) occurred, the investigator or subinvestigator immediately reported it to the head of Kyoto University Hospital in accordance with the standard operating procedures of Kyoto University Hospital and the documented procedures for the study. In addition, the event was reported to the DSMC if necessary.

7.5.1.2.4.2 Adverse Events to be Reported to the PMDA and Reporting of the Events

If a serious adverse event occurred, the investigator promptly considered the predictability of the event and the causal relationship to the investigational drug to determine whether it should be reported within 7 or 15 days (based on Article 273 of the Act on Securing Quality, Efficacy and Safety of Pharmaceuticals, Medical Devices, Regenerative and Cellular Therapy Products, Gene Therapy Products, and Cosmetics). If deemed necessary, the sponsor investigator reported the event to the Pharmaceuticals and Medical Devices Agency (hereinafter referred to as PMDA).

7.5.1.2.4.3 Report to the Hospital Head (Second and Subsequent Reports)

The investigator promptly reported any new information obtained after the first report [Section 7.5.1.2.4.1 ,

"Report to the Hospital Head, etc. (First Report)"] to the head of Kyoto University Hospital in writing. The sponsor investigator additionally reported the information to the PMDA if necessary.

7.5.2 Appropriateness of Measurements

Since KUS121 was administered to humans for the first time in this study, it was the most important to gather safety information; therefore, the primary endpoint was adverse events and adverse drug reactions.

Since the investigational drug is intravitreally administered to act locally, the amount of KUS121 distributed to non-ocular organs through general circulation is expected to be minimal. The pharmacokinetics of KUS121 was evaluated to confirm sufficiently low systemic exposure and estimate the kinetics of KUS121 in the vitreous body and retina/choroid.

The efficacy endpoints were decimal visual acuity, ETDRS visual acuity, and GP, which are generally used in the ophthalmologic field. In addition, the retinal sensitivity was selected to assess the retinal function closely, and the retinal thickness measured by OCT was selected to assess retinal morphological changes.

7.5.3 Definitions of Endpoints

Since the efficacy was a secondary objective of this study, no primary endpoint was specified for the efficacy.

7.5.3.1 Primary Endpoint

7.5.3.1.1 Adverse Events and Adverse Drug Reactions

In this study, an adverse event was defined as any unfavorable or unintended sign (including abnormal laboratory changes), symptom, or disease in a subject enrolled in the study and administered the investigational drug, whether or not it is related to the investigational drug or administration procedure.

Any adverse event for which a causal relationship to the investigational drug could not be ruled out was handled as an adverse drug reaction.

7.5.3.2 Secondary Endpoints

7.5.3.2.1 Incidences of Administration Procedure-Related Adverse Events

Any adverse event for which a causal relationship to the administration procedure could not be ruled out was handled as an adverse drug event , whether or not it was related to the investigational drug.

7.5.3.2.2 Pharmacokinetics of KUS121

For plasma KUS121 concentrations in the KUS121 25 μg and 50 μg groups, the changes at 2 and 4 hours post-dose on Day 1, at pre-dose and 2 and 4 hours post-dose on Day 2, and at pre-dose and 2, 4, and 24 hours post-dose on Day 3 were assessed.

7.5.3.2.3 Decimal LogMAR Visual Acuity and ETDRS LogMAR Visual Acuity

For the decimal visual acuity and ETDRS visual acuity, the changes in logMAR visual acuity from baseline at Weeks 8 and 12 were assessed. In addition, the minimum measurement at Weeks 2, 4, 8, and 12 was assessed. Counting fingers, hand motion, light perception, and no light perception were defined as a logMAR visual acuity of 2.6, 2.9, 3.1, and 3.4, respectively.

7.5.3.2.4 Proportion of Subjects with Improvement in Decimal LogMAR Visual Acuity and ETDRS LogMAR Visual Acuity

For the decimal visual acuity, the changes in decimal logMAR best-corrected visual acuity from baseline at Weeks 8 and 12 were assessed, with improvement defined as decrease of ≥ 0.3. With the number of subjects who received at least one dose of the investigational drug as the denominator, the proportion of subjects with improvement was determined. Also for the ETDRS visual acuity, the proportion of subjects with improvement was determined.

7.5.3.2.5 Proportion of Subjects with a Decimal Visual Acuity of ≥ 0.02

With the number of subjects who received at least one dose of the investigational drug as the denominator, the proportion of subjects with a decimal best-corrected visual acuity of ≥ 0.02 at Week 12 was determined.

7.5.3.2.6 Proportion of Subjects with a Decimal Visual Acuity of ≥ 0.05

With the number of subjects who received at least one dose of the investigational drug as the denominator, the proportion of subjects with a decimal best-corrected visual acuity of ≥ 0.05 at Week 12 was determined.

7.5.3.2.7 Proportion of Subjects with a Decimal Visual Acuity Better Than 0.1

With the number of subjects who received at least one dose of the investigational drug as the denominator, the proportion of subjects with a decimal best-corrected visual acuity of > 0.1 (not including 0.1) at Week 12 was determined.

7.5.3.2.8 Number of Letters Read on the ETDRS Chart

For the ETDRS visual acuity, the changes in number of letters read from baseline at Weeks 8 and 12 were assessed. In addition, the maximum measurement at Weeks 2, 4, 8, and 12 was assessed.

7.5.3.2.9 Visual Field Area Measured by GP

The change in isopter (V4e, I4e, and I2e) area from baseline at Week 12 was assessed.

7.5.3.2.10 Visual Field Score Measured by GP

The changes in visual field score (VFS)[23] and Esterman disability score (EDS)[24] for the V4e isopter from baseline at Week 12 were assessed.

7.5.3.2.11 Retinal Sensitivity

The change in retinal sensitivity at eight central points and surroundings from baseline at Week 12 was assessed.

7.5.3.2.12 ERG Latency and Amplitude

The flicker ERG latency and amplitude were assessed at Weeks 4 and 12 versus baseline. In addition, the changes in the difference in latency from the fellow eye at baseline and the amplitude ratio relative to the fellow eye at baseline over time were assessed.

7.5.3.2.13 Retinal Thickness

The changes in total retinal thickness at ≤ 1 mm, 1-3 mm, and 3-6 mm from the central fovea measured by OCT (SPECTRALIS raster scan) from baseline at Weeks 8 and 12 were assessed. In addition, the changes in the difference in total retinal thickness from the fellow eye at baseline over time were assessed.

7.5.3.2.14 Comparison of Retinal Circulation Time or Time of Maximal Venous Filling versus MBR

Retinal circulation time or time of maximal venous filling measured by fluorescein angiography was compared with MBR measured by laser speckle flowgraphy at baseline and on Day 6.

7.5.4 Drug Concentration Measurements

Plasma KUS121 concentrations in subjects were measured by Shin Nippon Biomedical Laboratories, Ltd. [see Section 4. (3)] using LC/MS/MS in accordance with a measurement protocol [plasma KUS121 concentration measurement study for the Phase I/II study of KUS121 for safety and efficacy evaluation in non-arteritic central retinal artery occlusion after intravitreal administration for 3 days (Protocol No.: iACT-15014)]. Plasma specimens collected from subjects were stored frozen until the measurement.

7.6 Data Quality Assurance

The sponsor investigator performed quality control and quality assurance in accordance with the operating procedures under the organization described in Section 4. , "Investigators and Study Administrative Structure" to ensure that the study was conducted in compliance with the protocol as well as applicable regulatory requirements, including the Act on Securing Quality, Efficacy and Safety of Pharmaceuticals, Medical Devices, Regenerative and Cellular Therapy Products, Gene Therapy Products, and Cosmetics and the MHW Ordinance on GCP.

7.6.1 Data and Safety Monitoring Committee (DSMC)

The sponsor investigator founded the DSMC to supervise whether the study was being conducted safely and appropriately [see Section 4. (4)]. If necessary, the DSMC discussed any issue listed below and made a recommendation to the sponsor investigator.

- Progression from the low dose to the high dose
- A significant safety problem occurs.
- The sponsor investigator determines that any other issue should be discussed by the DSMC.

Details were separately specified in documented procedures for the DSMC.

7.6.2 Study Quality Control

The investigator and the study site cooperated with the study monitor in direct inspection of all study-related records such as source documents.

The data management manager at the data center managed case report forms and data.

7.6.3 Study Quality Assurance

To assure the reliability of materials collected in the course of this study, audit was performed from the perspective of an outsider to investigate whether the study was conducted in accordance with the MHW Ordinance on GCP and the protocol. Audit certificates are provided in Appendix 16.1.8.

7.7 Statistical Methods Planned in the Protocol and Determination of Sample Size

7.7.1 Statistical and Analytical Plans

The items and contents of statistical analyses were detailed in a statistical analysis plan, and the statistical analysis plan is provided in Appendix 16.1.9.

7.7.1.1 Analysis Sets

7.7.1.1.1 All Enrolled Subjects

All enrolled subjects were defined as all subjects enrolled in the study.

7.7.1.1.2 Safety Analysis Set

The safety analysis set (SAS) was defined as the population of subjects who were enrolled in the study and received at least one dose of the investigational drug.

7.7.1.1.3 Efficacy Analysis Set

The efficacy analysis set (EAS) was defined as the population of subjects who were enrolled in the study

and received the investigational drug as specified. Any subject who was found to have major protocol violation or GCP violation after registration or found to be ineligible for the study after registration were excluded from the EAS.

7.7.1.2 Handling of Data

7.7.1.2.1 Handling of Subjects

The sponsor investigator and the statistical analysis manager discussed any problem concerning the analysis set for an enrolled subject to decide how to handle the relevant subject and documented the details of the problem, including the date of decision. For any problem that would affect the evaluation of the endpoints (conclusions), the DSMC was consulted.

7.7.1.2.2 Handling of Data

In principle, data were handled as described below for tabulation and analysis. However, any questionable data was discussed by the sponsor investigator and the statistical analysis manager to decide how to handle it.

- Any missing test or measurement data is not imputed, but is handled as missing.
- If there are multiple observations in a given period, one collected on the day closest to the specified date is used.

7.7.1.3 Statistical Analyses

In this study, statistical analyses were planned as described below. Technical details of analysis methods and detailed procedures for statistical analyses were separately specified in a statistical analysis plan (see Appendix 16.1.9). These statistical analyses were performed using SAS version 9.4 or newer. Any change to the statistical analysis plan was detailed in the clinical study report.

7.7.1.3.1 Demographic and Other Baseline Characteristics

(1) Composition of subjects

For each analysis set, the disposition of subjects and the number of ineligible subjects were calculated and shown in a flow chart.

(2) Distribution and Summary Statistics of Subject Characteristics

In the SAS, background factors were tabulated, and the distribution of each factor potentially affecting the results was checked.

7.7.1.3.2 Primary Analysis

In the SAS, the incidences of adverse events and adverse drug reactions following study treatment were tabulated for each dose group. In addition, the proportion of subjects who experienced an adverse event and the proportion of subjects who experienced an adverse drug reaction were calculated for each dose group, and

the CI for the proportion was calculated using an exact binomial method. In addition, the same analyses were performed by seriousness and severity.

7.7.1.3.3 Secondary Analyses

7.7.1.3.3.1 Analysis of Safety

In the SAS, each endpoint was analyzed as described below.

(1) Incidences of adverse events and adverse drug reactions by adverse event term

By adverse event term, the incidences of adverse events and adverse drug reactions following study treatment were tabulated for each dose group, and the proportion of subjects who experienced an adverse event and the proportion of subjects who experienced an adverse drug reaction were calculated. In addition, the 95% CI for the proportion was calculated using an exact binomial method. In addition, the same analyses were performed by seriousness and severity.

(2) Incidence of administration procedure-related adverse events

By adverse event term, the incidences of administration procedure-related adverse events following study treatment were tabulated, and the proportion of subjects who experienced an adverse event was calculated. In addition, the 95% CI for the proportion was calculated using an exact binomial method.

(3) Pharmacokinetics of KUS121

For the plasma concentration of the investigational drug in the low dose and high dose groups, main pharmacokinetic parameters were calculated.

7.7.1.3.3.2 Analysis of Efficacy

In the EAS, each endpoint was analyzed as described below.

Subjects with patent cilioretinal artery were assessed separately from those without patent cilioretinal artery.

(1) Decimal logMAR visual acuity and ETDRS logMAR visual acuity

For the decimal visual acuity and ETDRS visual acuity at baseline, Week 8, and Week 12, mean, SD, median, minimum, maximum, and two-sided 95% CI for logMAR visual acuity were summarized for each dose group. For the minimum measurement at Weeks 2, 4, 8, and 12, the same analyses were performed. In addition, a time course in each subject was plotted.

(2) Proportion of subjects with improvement in decimal logMAR visual acuity and ETDRS logMAR visual acuity

For the decimal visual acuity and ETDRS visual acuity at baseline, Week 8, and Week 12, the proportion of subjects with improvement defined as decrease in logMAR visual acuity of ≥ 0.3 and its exact binomial 95% CI were calculated for each dose group.

(3) Proportion of subjects with a decimal visual acuity of ≥ 0.02

With the number of subjects who received at least one dose of the investigational drug as the denominator, the proportion of subjects with a decimal best-corrected visual acuity of ≥ 0.02 at Week 12 and its exact binomial 95% CI were determined for each dose group.

(4)　Proportion of subjects with a decimal visual acuity of ≥ 0.05

With the number of subjects who received at least one dose of the investigational drug as the denominator, the proportion of subjects with a decimal best-corrected visual acuity of ≥ 0.05 at Week 12 and its exact binomial 95% CI were determined for each dose group.

(5)　Proportion of subjects with a decimal visual acuity better than 0.1

With the number of subjects who received at least one dose of the investigational drug as the denominator, the proportion of subjects with a decimal best-corrected visual acuity better than 0.1 at Week 12 and its exact binomial 95% CI were determined for each dose group.

(6)　Number of letters read on the ETDRS chart

For the number of letters read on the ETDRS chart at baseline and Week 12, mean, SD, median, minimum, maximum, and two-sided 95% CI were described for each dose group.

(7)　Visual field area measured by GP

For the change in isopter (V4e, I4e, and I2e) area from baseline at Week 12, mean, SD, median, minimum, maximum, and two-sided 95% CI were summarized for each dose group.

(8)　Visual field score measured by GP

For the VFS[23)] and EDS[24)] for the V4e isopter at baseline and Week 12, mean, SD, median, minimum, maximum, and two-sided 95% CI were summarized for each dose group.

(9)　Retinal sensitivity

For the retinal sensitivity measured by MP-3 at eight central points and surroundings at baseline and Week 12, mean, SD, median, minimum, maximum, and two-sided 95% CI were summarized for each dose group.

(10)　ERG latency and amplitude

For the latency and amplitude at each site as well as the difference (ratio for the amplitude) from baseline latency and amplitude in the fellow eye at baseline, Week 4, and Week 12, mean, SD, median, minimum, maximum, and two-sided 95% CI were summarized for each dose group.

(11)　Retinal thickness

For the total retinal thickness at each site as well as the difference from baseline total retinal thickness in the fellow eye at baseline, Week 8, and Week 12, mean, SD, median, minimum, maximum, and two-sided 95% CI were summarized for each dose group.

(12)　Comparison of retinal circulation time or time of maximal venous filling versus MBR

For the relationship between retinal circulation time or time of maximal venous filling and MBR at baseline and on Day 6, Pearson correlation coefficient and Spearman rank correlation coefficient were calculated.

7.7.1.3.4 Interim Analyses

No interim analysis was performed.

7.7.2 Determination of Sample Size

In this study, the target sample size was 9 subjects enrolled in the study who received at least one dose of the investigational drug. If no safety problem was detected in 3 subjects treated at the low dose, 6 subjects were treated at the high dose (Section 7.4.1.4 , "Decision to Progress to the Next Cohort").

[Rationale]

The annual incidence rate of central retinal artery occlusion is 0.7 to 1.8 per 100,000 persons[14)-16)], with the estimated number of new patients being approximately 1300 nationwide and approximately 20 in Kyoto Prefecture (population, 2.61 million people). In fact, 6 patients with central retinal artery occlusion visited the Department of Ophthalmology, Kyoto University Hospital during one year from October 2014 to September 2015, and all but 2 patients were ineligible for the present study due to a prolonged time (> 48 hours) after the onset at the initial visit or other reasons. While subjects will be recruited at nearby hospitals and eye clinics in Kyoto City, the maximum possible sample size is a maximum of 9 subjects per year, and this target sample size was determined based on the number of patients and the feasibility of recruitment.

Furthermore, the investigational drug, which has never been administered to humans, should be administered at the high dose only after confirming the safety at the low dose. Accordingly, the sample size of 3 subjects for the low dose group and 6 subjects for the high dose group was determined.
